# Oscillatory Stiffness Versus Creep–Recovery Response of Recycled High-Density Polyethylene/Waste Engine Oil Modified Bitumen

**DOI:** 10.3390/polym18212785

**Published:** 2026-11-07

**Authors:** Hassan Shuaibu Abdulrahman, Michael Chiahemba Iorpenda, Muhammed Fatih Yentimur, Ali Almusawi, Mustafa Albdairi

**Affiliations:** 1 Department of Civil Engineering, Federal University of Technology Minna, Minna P.M.B. 65, Niger State, Nigeria; abdul.hassan@futminna.edu.ng (H.S.A.); iorpendamcmen82@st.futminna.edu.ng (M.C.I.); 2 Department of Civil Engineering, Faculty of Engineering and Architecture, Recep Tayyip Erdoğan University, Rize 53100, Türkiye; 3 Department of Civil Engineering, Faculty of Engineering, Çankaya University, Ankara 06815, Türkiye; ali.almusawi@cankaya.edu.tr (A.A.); c2290007@student.cankaya.edu.tr (M.A.)

**Keywords:** recycled high-density polyethylene, waste engine oil, polymer-modified bitumen, multiple stress creep recovery, fractional viscoelasticity, springpot, nonlinear viscoelasticity, Cox–Merz rule, activation energy, black diagram

## Abstract

Recycled high-density polyethylene (HDPE) and waste engine oil (WEO) can be combined to modify bitumen, but the high-temperature performance of such binders is often judged from oscillatory measurements alone. This study compares the oscillatory and creep–recovery responses of two long-term-aged HDPE/WEO binders with the same total modifier content but different HDPE:WEO ratios with those of the unmodified base bitumen. Dynamic shear rheometer (DSR) temperature sweeps and multiple stress creep recovery (MSCR) tests, also performed on the DSR, were combined with fractional viscoelastic modeling. Both blends reduced the non-recoverable creep compliance by a factor of 3.8–7.3, so the modification improved the resistance to permanent deformation. In oscillation, however, the blends became more elastic as the temperature increased, whereas in creep, they recovered almost no strain and crept 2–122 times more than predicted from their oscillatory response; the base bitumen was predicted within a factor of 1.6. The creep response of the blends was also four to five times more temperature-sensitive than their oscillatory response. A three-parameter fractional Maxwell model described all creep–recovery cycles and captured the power-law recovery of the blends better than the Burgers model. The results are consistent with an HDPE-rich structure that dominates the oscillatory response but breaks down at MSCR strains, so oscillatory stiffness overstates both the elastic character and the magnitude of the improvement achieved by the binders tested.

## 1. Introduction

Post-consumer plastics and used lubricating oils are two of the most persistent waste streams of modern transport and consumer economies. Bitumen modification offers an outlet for both, because asphalt binder is used in very large volumes relative to the few percent of modifier normally required [1,2,3]. Among recycled plastics, polyolefins, and high-density polyethylene (HDPE) in particular, have received sustained attention as low-cost plastomeric modifiers that raise the high-temperature stiffness of the binder [4,5,6], and the same route is being explored for other polymeric wastes such as disposable medical gloves [7]. Polyethylene (PE) is semicrystalline and largely insoluble in bitumen; it modifies the binder as a swollen, dispersed phase rather than by chemical reaction [8,9,10], and its tendency to separate during hot storage is one of the main obstacles to its use [11,12,13,14].

Waste engine oil (WEO) is often combined with polyethylene for two reasons. Used alone, it supplies light, maltene-like fractions that lower the stiffness of the binder and raise its phase angle, and large doses reduce high-temperature resistance [15,16,17]. In polyolefin-modified binders it also acts as a compatibilizer that helps the polymer swell and disperse: used engine oil increased the amount of plastic waste that could be dissolved in a hard bitumen [18], WEO reduced the aggregation of waste PE seen by fluorescence microscopy [19], and similar effects of oils on dispersion and storage stability have been reported for other polymer systems [20,21]. Only a few binder-level studies combine PE with engine oil [19,22,23], and in none of them was the polymer: oil ratio varied at constant total modifier content, so the effect of the ratio cannot be separated from that of the total dosage.

The high-temperature performance of such binders is usually judged by oscillatory dynamic shear rheometer (DSR) parameters such as |G*|/sin δ, whereas the multiple stress creep recovery (MSCR) test, whose non-recoverable creep compliance J_nr_ correlates better with mixture rutting, is now the preferred measure for modified binders [24,25,26,27,28]. For PE binders the two measures often diverge. In oscillation, PE binders show a phase angle that stops increasing or even decreases with temperature, a low-frequency modulus plateau and large gains in the high-temperature grade at modest dosages [10,12,29,30,31]. In MSCR the same type of binder shows low recovery at 3.2 kPa, high stress sensitivity and J_nr_ reductions that are much smaller than the grade gains would suggest [22,23,32]. In the interlaboratory program of RILEM TC 279-WMR the recovery of one PE blend at 3.2 kPa ranged from 6% to 96% between laboratories, and undissolved particles were suspected to dominate the measurement once the matrix became fluid [12]. Supplementary Tables S1–S3 and Figures S1 and S2 summarize the binder-level evidence on these points.

The divergence has a plausible mechanical origin. The linear viscoelastic range of PE binders extends only to a few percent strain [29,33], whereas MSCR strains in soft binders reach tens of percent at 0.1 kPa and thousands of percent at 3.2 kPa [27], and the Cox–Merz rule, which equates oscillatory and steady-flow viscosities in linear fluids [34], fails for HDPE binders [30,35]. Filled elastomers offer an analogy: carbon-black-filled rubbers have a high storage modulus at low strain amplitudes that falls sharply as the amplitude increases, because the network of filler–filler contacts breaks down progressively, which is known as the Payne effect [36]. If HDPE-rich domains in bitumen form load-bearing contacts in a similar way, a binder could appear highly elastic in an oscillatory test yet flow almost freely under MSCR loading. Two tools can test this idea quantitatively. The creep response of a linear binder can be predicted from its oscillatory response, as shown for unmodified and elastomer-modified binders [37], and fractional viscoelastic models built from the springpot element describe the broad, power-law relaxation of modified binders with few parameters [38,39,40,41,42,43].

Four gaps follow from this literature. First, the oscillatory and creep responses of PE binders have not been reconciled quantitatively, by predicting the MSCR creep from the oscillatory data and measuring the departure from that prediction. Second, it is not known whether the decreasing phase angle reflects a binder-scale structure that breaks down at MSCR strains or particle contacts at the scale of the measuring gap. Third, the effect of the polymer:oil ratio at constant total modifier content has not been examined in aged PE–engine-oil binders. Fourth, fractional models have been applied only to elastomer-, rubber- and acid-modified binders, and their predictive reliability has rarely been tested beyond the data to which they were fitted.

This study addresses these gaps for two HDPE/WEO blends with the same total modifier content of 12 wt% but HDPE:WEO ratios of 2:1 and 1:1, prepared with a 60/70 penetration-grade bitumen and compared with the unmodified bitumen after long-term aging in the pressure aging vessel (PAV). The objectives were to quantify the departure of the blends from linear viscoelasticity by predicting their MSCR creep from the oscillatory data and by comparing creep and complex viscosities; to derive indicators of structural breakdown from the MSCR data alone; to estimate the effective volume fraction of the HDPE-rich phase and the strain at which it breaks down; to compare the two HDPE:WEO ratios in both tests; and to model the creep–recovery cycles with fractional and Burgers models whose predictive reliability is checked by cross-validation. Unlike previous studies of polyolefin-modified binders, which reported oscillatory and MSCR results side by side and noted their disagreement only qualitatively [10,12,19,23], the present study treats the two tests as measurements of the same material at strains that differ by four to five orders of magnitude. Combining them with fractional modeling and an indirect structural analysis shows whether the discrepancy is real, how large it is, at what strain the structure responsible for it breaks down, and what the standard MSCR parameters measure once it has broken. The contribution of the work is therefore a simple, test-compatible framework for detecting when the oscillatory response of a plastomer-modified binder misrepresents its large-strain behavior, together with a mechanistic reading of the standard MSCR parameters of such binders.

## 2. Materials and Methods

### 2.1. Materials

**Figure 1:**
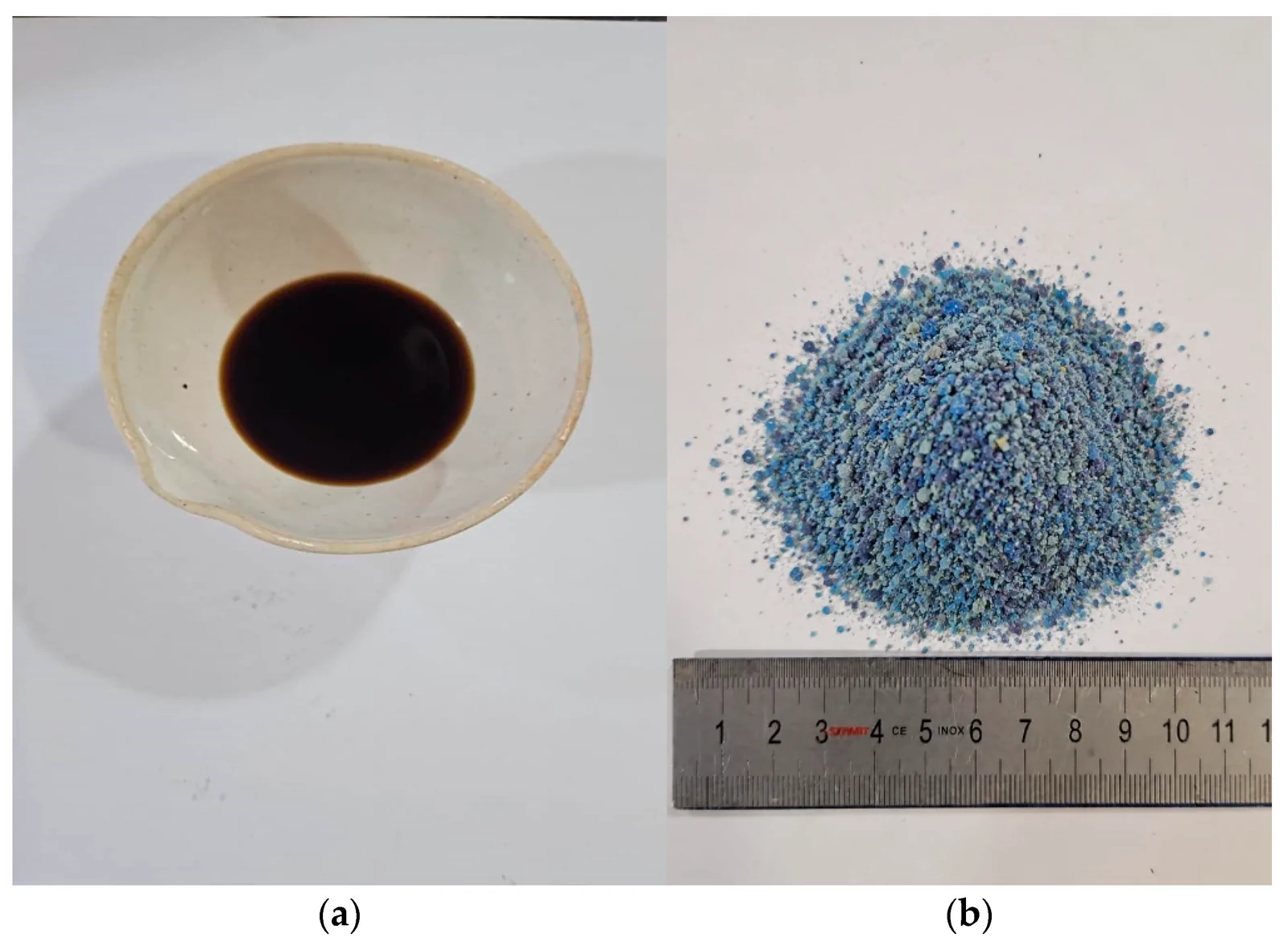
Recycled modifiers used in this study: (**a**) waste engine oil (WEO) drained from gasoline-powered passenger cars; (**b**) recycled HDPE particles (approximately 0.5–2 mm) obtained from post-consumer plastic chairs, with a ruler (cm) for scale.

A conventional 60/70 penetration-grade paving bitumen, sourced from TUNS Diamond Construction Company in Abeokuta, Ogun State, Nigeria, was used as the base binder and, without modification, served as the control binder (CONTROL). Its basic properties, taken from the supplier’s certificate of quality, are given in Table 1. WEO drained only from gasoline-powered passenger cars, with no oil from diesel or generator engines, was collected from a local automotive workshop in Abeokuta and stored within a period of 2–3 weeks before use; it was filtered through a 100 μm sieve and used without chemical treatment. Recycled HDPE, obtained mainly from post-consumer plastic chairs from recycling sources in Abeokuta, was cleaned and ground into particles of approximately 0.5–2 mm. The WEO had a flash point of 110 °C and a fire point of 135 °C in the Cleveland open cup (ASTM D92) [44], and the characteristics of both modifiers are summarized in Table 2. The two modifiers are shown in Figure 1. The laboratory work was carried out at the Centre for Advanced Pavement Technology, Nigerian Building and Road Research Institute (NBRRI), Abuja, Nigeria.

### 2.2. Binder Preparation

Two modified binders were studied. W4H8 contained 4 wt% WEO and 8 wt% HDPE, and W6H6 contained 6 wt% WEO and 6 wt% HDPE, both by mass of base bitumen (Table 3). The two blends therefore carry the same total modifier content of 12 wt% but differ in their HDPE:WEO ratio, 2:1 and 1:1. The two compositions were selected from a preliminary set of 16 unaged blends, with WEO and HDPE contents of 2, 4, 6 and 8 wt% each, by grey relational analysis of penetration, softening point and viscosity; W4H8 and W6H6 had the highest grey relational grades among the blends with a total modifier content of 12 wt%, which allowed the effect of the HDPE:WEO ratio to be studied at constant total content. Both modifiers were incorporated into the hot base bitumen by wet blending at 160 ± 5 °C, well above the melting range of HDPE, using a high-shear mixer (L5M-A, Silverson Machines Ltd., Chesham, UK). WEO was added first and mixed at 500 rpm for 5 min, after which HDPE was added and mixed at 4000 rpm for 60 min. This protocol was designed to achieve a fine dispersion of the polymer and thereby improve storage stability. Blending above the melting range of recycled HDPE under high shear, with the oil added first as a compatibilizer, has been shown to give a fine and uniform dispersion of polyethylene in bitumen and to reduce phase separation [13,19,30,45]. Polyethylene-modified binders nevertheless remain prone to separation during hot storage [12,14], and storage stability was not measured in this study. Because blending was carried out above the melting range of HDPE, the initial size of the ground particles affects the time needed to melt and disperse them rather than the final structure, which forms on cooling and is characterized indirectly in Section 3.11.

### 2.3. Aging

The binders were aged in the PAV (Infratest Prüftechnik GmbH, Brackenheim, Germany) in accordance with AASHTO R 28 of the American Association of State Highway and Transportation Officials (AASHTO) [46] at a pressure of 2.1 MPa and a temperature of 100 °C for 20 h, without prior rolling thin-film oven test (RTFOT) conditioning, with the binders placed in the standard stainless-steel pans of the PAV pan holder. The standard sequence of AASHTO R 28 includes short-term aging in the RTFOT before PAV aging, because the RTFOT residue represents the starting state for long-term aging [47,48]. In this study, PAV aging was applied directly to the unaged binders, as in earlier studies of the long-term oxidative aging of bitumen [49] and of thermoplastic-modified binders [50], so that all binders were compared in a single long-term-aged state. All binders, including the control, were aged with the same protocol, so the comparisons made in this paper refer to the same aging state, and the oscillatory and creep responses were measured on the same aged material. This choice has two consequences, which limit the comparison with standard practice. First, because short-term aging was omitted, this state is expected to be somewhat less oxidized than that obtained with the standard RTFOT and PAV sequence. Second, the RTFOT step at 163 °C would remove part of the volatile fraction of the WEO, which direct PAV aging at 100 °C may not, so the blends may retain more of the light oil than after the standard sequence; this would affect their absolute stiffness more than the comparison between the oscillatory and creep responses, which were measured on the same aged material. All results reported in this paper refer to PAV residues, which represent a long-term-aged binder relevant to the performance of pavements in hot climates. The only exception is the base bitumen tested before aging with the same oscillatory and MSCR protocols, which is used solely as an additional linear reference (Section 3.2 and Section 3.4; Table S9). AASHTO T 350 and M 332 specify the MSCR test and its traffic criteria for short-term-aged RTFOT residue [24,25]; here the MSCR test is applied to PAV residues to compare the binders and analyze their response, and the traffic levels derived from it (Section 3.2) are therefore indicative. Because PAV residues are more oxidized than RTFOT residues, these traffic levels are likely to be higher than those that the same binders would obtain after RTFOT aging alone, and they should not be used for specification purposes.

### 2.4. Oscillatory Testing

Oscillatory measurements were performed with a Kinexus DSR+ dynamic shear rheometer (NETZSCH-Gerätebau GmbH, Selb, Germany) controlled with rSpace software (v2.00) using 25 mm parallel plates and a 1 mm gap at an angular frequency of 10 rad/s (1.59 Hz) and temperatures of 52–82 °C in 6 °C steps. The strain amplitude was 0.01%, which lies well within the linear viscoelastic range of bitumen [51]. For polyethylene-modified binders, amplitude sweeps have shown the onset of nonlinearity at strains of about 1.4–4.1% [33], more than one hundred times the amplitude used here. The oscillatory data therefore represent the linear response required for the linear prediction of Section 2.7, and they are not used to grade the binders [52,53].

### 2.5. Multiple Stress Creep Recovery Testing

MSCR tests were carried out on the same dynamic shear rheometer according to AASHTO T 350 [24] with 25 mm plates at 64, 70, 76 and 82 °C. AASHTO M 332 specifies that the MSCR test be performed at the high climatic pavement temperature of the project, with traffic accounted for through the limits on J_nr,3.2_ rather than by testing at a higher grade [25]. Because the binders were not designed for a specific project, they were tested at four standard high-temperature grades in 6 °C steps, from 64 °C, a grade commonly specified for pavements in hot climates [54], to 82 °C, the highest standard grade and the upper limit of the oscillatory measurements (Section 2.4). This allows the indicative traffic level of each binder to be read at any of these grades (Section 3.2) and the temperature dependence of the creep response to be analyzed (Section 2.9). Each test comprised ten creep–recovery cycles at 0.1 kPa followed by ten cycles at 3.2 kPa, each cycle consisting of 1 s of creep and 9 s of recovery; strain was sampled every 0.1 s. For each cycle the peak strain ε_c_ at the end of creep and the residual strain ε_r_ at the end of recovery were obtained relative to the start of the cycle, and

J_nr_ = ε_r_/σ_0_ (1)

%R = 100(ε_c_ − ε_r_)/ε_c_ (2)

J_nr,diff_ = 100(J_nr,3.2_ − J_nr,0.1_)/J_nr,0.1_ (3)

were averaged over the ten cycles at each stress σ_0_. The recoverable strain ε_c_ − ε_r_ was also recorded for each stage. Traffic levels were assigned with the AASHTO M332 limits on J_nr,3.2_ of 4.5, 2.0, 1.0 and 0.5 kPa^−1^ (Standard, Heavy, Very Heavy, Extremely Heavy) [25], and the temperatures up to which each limit was met were obtained by log-linear interpolation of J_nr,3.2_ with temperature. Recovery at 3.2 kPa was compared with the informational M332 curve %R_3.2_ = 29.371 J_nr,3.2_^−0.2633^ (valid for J_nr,3.2_ ≤ 5 kPa^−1^) [25]. One specimen was tested per binder and temperature.

### 2.6. Fractional and Classical Creep–Recovery Modeling

For each binder, temperature and stress level, the ten cycles were averaged point by point to give a mean cycle of 100 points (10 in creep and 90 in recovery). Three models were fitted. The three-parameter fractional Maxwell model (FM3) consists of a spring of modulus G in series with a springpot of constant c and order α [38,39,43], with creep compliance

J(t) = 1/G + t^α^/[c Γ(1 + α)] (4)

where Γ is the gamma function. For a 1 s stress step of magnitude σ_0_, Boltzmann superposition gives the strain within the cycle as

ε(t) = σ_0_ J(t) for 0 < t ≤ 1 s;   ε(t) = σ_0_[J(t) − J(t − 1)] for 1 s < t ≤ 10 s (5)

In the recovery phase the spring contribution cancels and only the springpot contributes, so recovery is controlled by α: for α < 1 the strain decays as a power law, and for α = 1 the springpot becomes a dashpot and the residual strain is entirely permanent. The four-parameter fractional model (FM4) replaces the spring with a second springpot (constant c_2_, order β ≤ α) [38,55]:

J(t) = t^α^/[c_1_ Γ(1 + α)] + t^β^/[c_2_ Γ(1 + β)] (6)

and the classical four-parameter Burgers model [56] is

J(t) = 1/G_0_ + t/η_0_ + (1/G_1_)[1 − exp(−t G_1_/η_1_)] (7)

Parameters were estimated in Python (v3.13) with SciPy (v1.18) and NumPy (v2.5) by nonlinear least squares (trust-region reflective algorithm) by nonlinear least squares (trust-region reflective algorithm) on residuals normalized by the peak strain, using 60 random starting points per fit and the bounds 0 ≤ α, β ≤ 1 and 10^1^ ≤ G ≤ 10^9^ Pa. Goodness of fit is reported as R^2^ and as the root-mean-square error normalized by the range of the data (NRMSE). Models were compared with the corrected Akaike information criterion (AICc) [57],

AICc = n ln(SSE/n) + 2k + 2k(k + 1)/(n − k − 1)(8)

where n is the number of points, k the number of parameters and SSE the sum of squared normalized residuals. A difference ΔAICc > 10 was taken as strong support for the model with the lower value [57].

The predictive reliability of the FM3 model was tested in three ways that use data not included in the fit. In a cycle split, the model was fitted to the mean of cycles 1–5 and used to predict the mean of cycles 6–10. In a phase split, it was fitted to the 1 s creep phase only and used to predict the 9 s recovery phase, which tests whether the parameters identified in loading describe unloading. In a temperature split, the springpot constant c of the 3.2 kPa fits was predicted at each temperature from the Arrhenius fit of the other three temperatures, and J_nr,3.2_ was computed from the predicted parameters. The Burgers model was subjected to the same cycle and phase splits for comparison. The sensitivity of the fitted parameters was assessed from their standard errors and correlations, obtained from the Jacobian at the solution, and from the change in J_nr_ and %R produced by a small relative change in each parameter.

### 2.7. Linear Prediction of Creep from Oscillatory Data (Λ Ratio)

If a binder is linear viscoelastic over the strains reached in MSCR, its creep compliance after 1 s of loading can be predicted from the oscillatory measurement at the same temperature [37]. Because one frequency was measured per temperature, two single-point calibrations were used to bracket the prediction. In the first, a springpot is matched exactly to the measured |G*| and δ at ω = 10 rad/s:

α_sp_ = δ/90°, c_sp_ = |G*|/ω^αsp^, J_lin_(1 s) = 1/[c_sp_ Γ(1 + α_sp_)] (9)

In the second, a Maxwell element (spring G_M_ in series with dashpot η_M_) is matched to the measured G′ and G″:

τ_M_ = G′/(ωG″), G_M_ = G′(1 + ω^2^τ_M_^2^)/(ω^2^τ_M_^2^), η_M_ = G_M_τ_M_, J_lin_(1 s) = 1/G_M_ + (1 s)/η_M_ (10)

The departure from linearity is then expressed by the ratio

Λ = J_meas_(1 s)/J_lin_(1 s) (11)

where J_meas_(1 s) = ε_c_/σ_0_ is the measured compliance at the end of creep. For a linear binder Λ ≈ 1. The springpot calibration assumes a constant phase angle across frequency and gives the higher Λ; the Maxwell calibration assumes a single relaxation time and gives the lower Λ. Because the creep time (1 s) lies only one decade from the oscillation time scale (1/ω = 0.1 s), the extrapolation in time is short. The comparison therefore rests on four assumptions: that the binder is linear up to the strain reached in creep, which is the hypothesis being tested; that its frequency dependence near 10 rad/s lies between the springpot and Maxwell limits; that a creep time of 1 s corresponds to an oscillation time of 0.1 s; and that the oscillatory and creep specimens of the same binder and temperature are equivalent. If the true frequency dependence lay outside the two limits, Λ could fall outside the reported range. For the control, however, Λ was close to 1 with both calibrations, which shows that the limits are adequate for a linear binder, whereas the values of the blends exceed them by one to two orders of magnitude.

### 2.8. Creep Viscosity and Cox–Merz Comparison

As a check that does not rely on a constitutive model, an apparent creep viscosity was obtained from the steady part of each mean creep phase,

η_creep_ = σ_0_/γ^.^, γ^.^ = dε/dt over 0.5 s ≤ t ≤ 1 s (12)

where γ^.^ is the slope of a linear regression of the mean cycle strain on time. For a linear, nearly Newtonian binder the Cox–Merz rule [34] implies η_creep_ ≈ |η*| = |G*|/ω, so the ratio |η*|/η_creep_ should be close to 1. The ratio was evaluated directly at ω = 10 rad/s (model-free) and, because the creep shear rates differed from 10 s^−1^, also at a frequency matched to γ^.^, with |η*| extrapolated by the springpot of Equation (9) as |η*(γ^.^)| = (|G*|/ω)(γ^.^/ω)^αsp−1^. The second value depends on the extrapolation and is reported only as a check. From the two stress levels of each MSCR test, a power-law flow index was also obtained,

n = 1 + ln(η_creep,3.2_/η_creep,0.1_)/ln(γ^.^_3.2_/γ^.^_0.1_) (13)

which equals 1 for a Newtonian liquid and is below 1 when the creep viscosity falls with increasing stress (stress softening). This index requires only MSCR data.

### 2.9. Temperature Sensitivity (Apparent Activation Energy)

The temperature sensitivity of the oscillatory and creep responses was described by an apparent Arrhenius activation energy E_a_ [56],

ln X = ln A + E_a_/(R_g_T) (14)

where X is |G*|, 1/J_nr,3.2_ or the springpot constant c of the 3.2 kPa fits, R_g_ = 8.314 J/(mol·K) and T is the absolute temperature. E_a_ was obtained by linear regression over 52–64 °C and 64–82 °C for |G*| and over 64–82 °C for the creep quantities, and is reported with its standard error and the coefficient of determination of the regression. Over such narrow ranges the Arrhenius form is an empirical summary rather than a statement about the underlying flow mechanism, and a poor fit is itself informative.

Thermorheological simplicity was tested on the recovery phase of the MSCR cycles. The recovered strain ε_c_ − ε(t′), where t′ is the time after unloading, was normalized by its value at t′ = 9 s and plotted against log t′, and each curve was shifted horizontally onto the 64 °C curve so as to minimize the root-mean-square (RMS) difference. For a thermorheologically simple binder, the recovery curves superpose and the recovery shift factor a_T_ equals the shift factor of viscous flow; for a power-law (springpot) element, a horizontal shift changes c but not α, so a temperature-dependent α also indicates a departure from simplicity [43].

### 2.10. Statistical Analysis (Bootstrap Confidence Intervals)

Uncertainty was quantified by a nonparametric bootstrap over the ten cycles of each stress level [58]. Cycles were resampled with replacement 2000 times, and J_nr_, %R, J_meas_(1 s) and Λ were recomputed for each resample; for the fractional Maxwell parameters the mean cycle was rebuilt from 300 resamples and refitted. Percentile 95% confidence intervals (CIs) are reported. These intervals describe the cycle-to-cycle variability within one specimen and do not include specimen-to-specimen variability (Section 6). Because each temperature was tested on a separate specimen, the scatter of J_nr,3.2_ about the Arrhenius fit of Section 2.9 also provides an upper-bound estimate of the variability between specimens, which includes any deviation of the data from the Arrhenius form.

Generative AI (Claude Opus 5.5, Anthropic, San Francisco, CA, USA) was used to assist in writing the Python (v3.13) scripts for model fitting and statistical analysis and in preparing the figures; all scripts, results and figures were checked by the authors.

### 2.11. Indirect Structural Analysis

To characterize the HDPE-rich phase without microscopy, two analyses of the existing data were added. First, the stiffening of the blends was interpreted with the Krieger–Dougherty model of suspension rheology [59,60], which relates the relative modulus G_r_ of a suspension to the effective volume fraction φ_eff_ of its dispersed phase:

G_r_ = (1 − φ_eff_/φ_max_)^−[η]φmax^ (15)

where [η] = 2.5 is the intrinsic viscosity of rigid spheres and φ_max_ = 0.64 is the volume fraction at random close packing. G_r_ was taken as the ratio of |G*| of each blend to that of the control at the same temperature, with the control modulus at 70–82 °C extrapolated from its Arrhenius fit over 52–64 °C. In creep, G_r_ was taken as the ratio of the apparent creep viscosity of each blend at 0.1 or 3.2 kPa to that of the control at 3.2 kPa, which behaves as a nearly Newtonian liquid (Section 3.5). The nominal volume fractions of HDPE, 0.058 for W6H6 and 0.077 for W4H8, were calculated from the compositions with densities of 1.040 g/cm^3^ for the bitumen (Table 1), 0.95 g/cm^3^ for HDPE and 0.88 g/cm^3^ for WEO. Because the model is derived for rigid spheres, φ_eff_ is an apparent value that indicates the volume over which the HDPE-rich phase acts mechanically.

Second, the strain at which the structure begins to break down was estimated from the ratio Λ. Because 1/Λ is the ratio of the linearly predicted to the measured compliance, it acts as a normalized modulus that equals 1 for a linear response. It was plotted against the strain of each test, taken as 0.01% for the oscillatory test and as the mean peak creep strain at 0.1 and 3.2 kPa, and fitted with the Kraus model used to describe the Payne effect in filled elastomers [36,61,62,63]:

1/Λ = g_∞_ + (1 − g_∞_)/[1 + (γ/γ_c_)^2m^] (16)

where γ_c_ is the critical strain, g_∞_ is the limiting normalized modulus at large strain and m = 0.5 is a fixed exponent. With only three strain levels per condition, the fit locates γ_c_ approximately, and it assumes that the oscillatory point is linear.

## 3. Results

### 3.1. Oscillatory Response and Black Diagram

**Figure 2:**
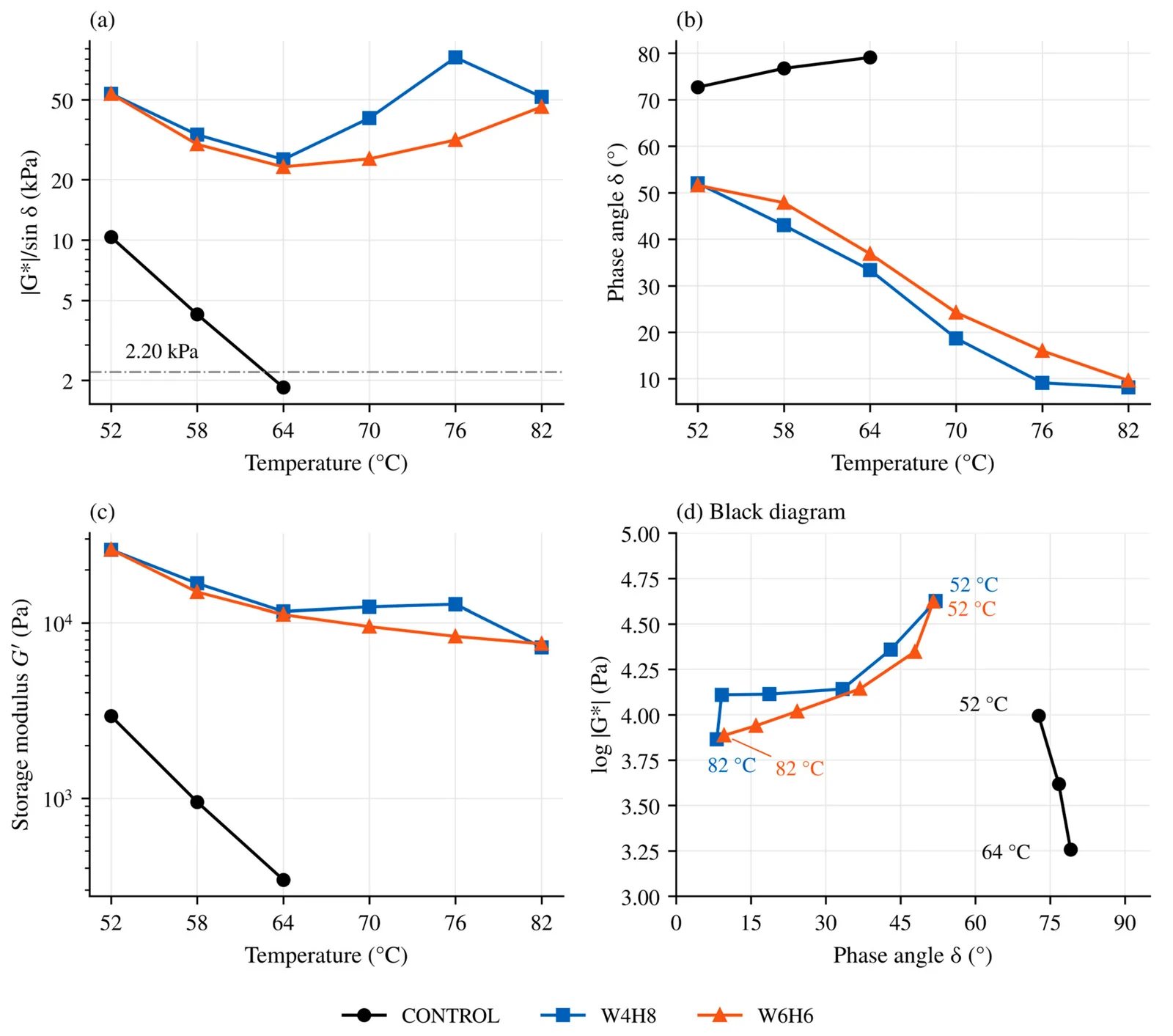
Oscillatory response of the PAV-aged binders at 10 rad/s: (**a**) |G*|/sinδ, with the 2.20 kPa reference value of AASHTO M320; (**b**) phase angle; (**c**) storage modulus; (**d**) black diagram, log |G*| versus phase angle (labels give the first and last temperature of each series). The control is plotted at 52–64 °C only, because at 70–82 °C its measured torques were close to the lower limit of the instrument (Section 3.1).

Figure 2 summarizes the oscillatory data, and the numerical values are given in Table S4. The aged control behaved as an ordinary unmodified binder: between 52 and 64 °C its |G*| fell from 9.88 to 1.81 kPa and its phase angle rose from 72.7° to 79.1°. The control data at 70–82 °C are not reported, because the measured torques of 0.07–0.25 μN·m were close to the lower limit of the instrument.

The modified binders behaved very differently. At 52 °C both had |G*| ≈ 42 kPa and δ ≈ 52°, a stiffer but still predominantly viscoelastic response. With increasing temperature, however, δ fell steadily, reaching 8.2° for W4H8 and 9.6° for W6H6 at 82 °C (Figure 2b), while G′ changed little above 64 °C and remained between 7.3 and 13 kPa (Figure 2c). The two blends were almost indistinguishable at 52–64 °C; above 64 °C, W4H8 had the lower phase angle and the higher, nearly constant G′, which stayed between 12.3 and 12.7 kPa at 70–76 °C. A phase angle that decreases with temperature and a storage modulus that becomes nearly temperature-independent are the opposite of the behavior of the bitumen matrix, whose δ approaches 90° as it softens. They indicate that, in the oscillatory test, the response at high temperature is dominated by a stiff, largely elastic structure associated with the HDPE phase rather than by the bitumen. As a consequence |G*|/sinδ of the modified binders passed through a minimum near 64 °C and then increased with temperature (Figure 2a). This non-monotonic behavior is a further reason why these data cannot be used to assign a high-temperature grade to the modified binders.

The black diagram (Figure 2d) summarizes these differences without reference to temperature. The control follows the steep, smooth path typical of unmodified bitumen [64], in which δ increases as |G*| decreases. The blends follow a quite different path: from 52 to 64 °C δ falls from 52° to 33–37° while |G*| decreases about threefold, and above 64 °C the curve turns almost horizontal, δ falling to 8–10° while |G*| stays within 7–14 kPa. A near-horizontal branch, in which the modulus stays nearly constant while the phase angle falls, is the black-diagram signature of an elastic structure that dominates the response once the matrix has softened, as observed for elastomer-modified binders at high polymer contents [64]. W4H8 shows this branch most clearly, with |G*| constant at 12.9–13.9 kPa between 64 and 76 °C. The normal force during these measurements was 0.21–0.30 N for the blends and 0.09–0.11 N for the control, so the blend specimens did not show the large normal forces that would indicate particles jammed between the plates (Section 4.1).

### 3.2. MSCR Response and Indicative Traffic Levels

**Figure 3:**
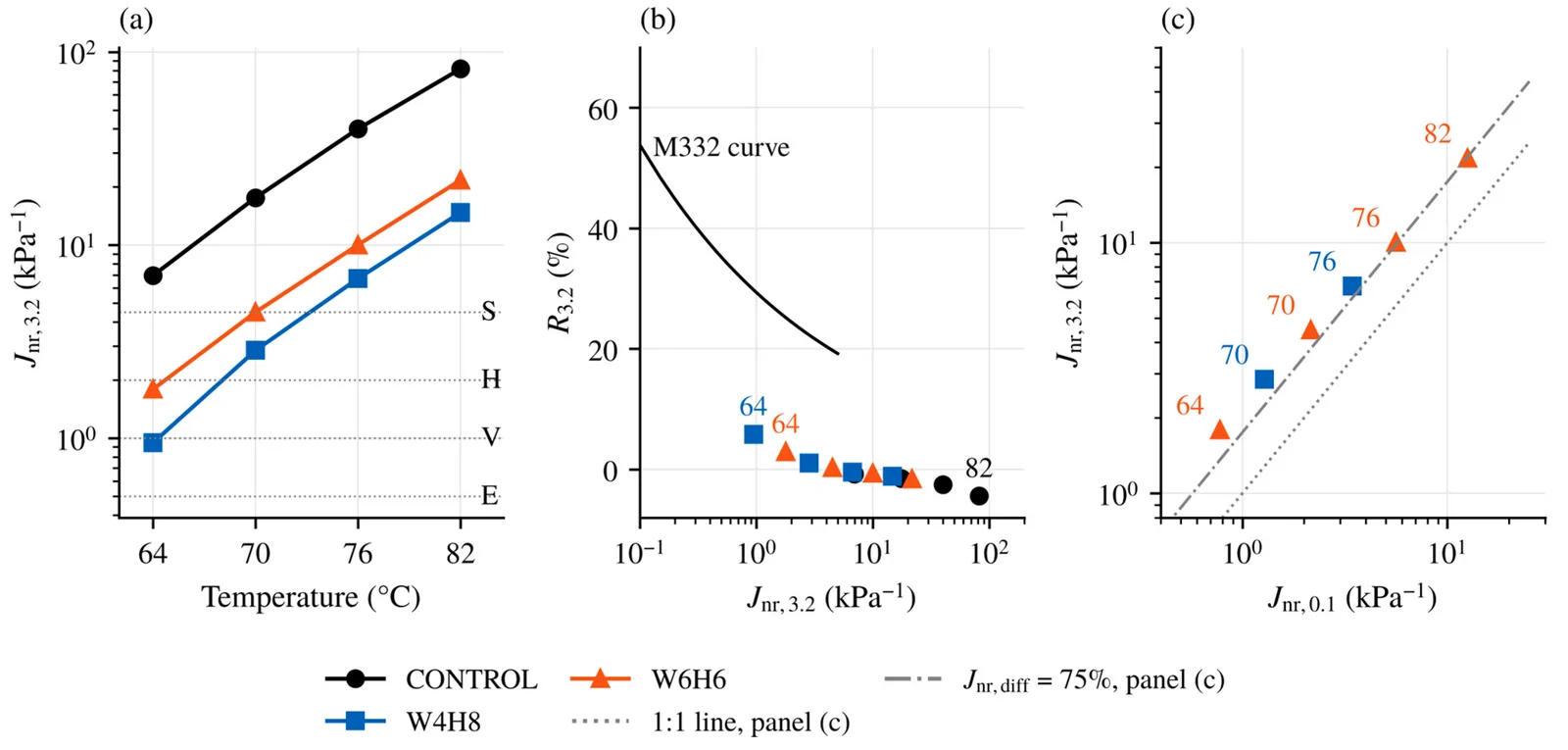
MSCR results of the PAV-aged binders: (**a**) J_nr,3.2_ versus temperature with the AASHTO M332 traffic-level limits (S, H, V, E); (**b**) %R_3.2_ versus J_nr,3.2_ with the M332 elastic-response curve; (**c**) J_nr,3.2_ versus J_nr,0.1_, with the 1:1 line and the 75% J_nr,diff_ limit. Selected labels give the test temperature in °C. Slightly negative values of %R_3.2_ correspond to recovery that is zero within the measurement resolution (Section 3.2).

Figure 3 shows the MSCR results, and the numerical values are given in Table S5. Both modified binders reduced J_nr,3.2_ substantially relative to the control at every temperature (Figure 3a): by a factor of 7.3 for W4H8 and 3.9 for W6H6 at 64 °C and by factors of 5.5 and 3.8 at 82 °C. W4H8 was the stiffer of the two blends at all temperatures, with J_nr,3.2_ 53–68% of that of W6H6, although both contain 12 wt% total modifier. On this measure the HDPE content, rather than the total modifier content, governed the improvement; the additional WEO in W6H6 partly offset the stiffening effect of the HDPE.

Three 0.1 kPa stages were not reported: that of the control, which was inconsistent with its 3.2 kPa stage, with compliances 18–540 times lower and a negative J_nr_ at 76 °C; that of W4H8 at 64 °C, where the recovery exceeded the creep strain; and that of W4H8 at 82 °C, where the between-cycle coefficient of variation in the peak strain was 31%. These stages were excluded as measurement effects rather than material behavior. For a nearly Newtonian binder such as the control, the compliances at the two stress levels should be similar, and a negative J_nr_, like a recovery that exceeds the creep strain, is physically impossible for a passive material. The same base bitumen tested before aging gave a normal 0.1 kPa stage, with J_nr_ at the two stress levels differing by only 14–28% (Table S9), which supports the conclusion that the stage of the aged control was affected by the measurement rather than by the material. The W4H8 stage at 64 °C combined the stiffest binder with the lowest stress, so its creep strain was the smallest of the test program and a small drift during recovery outweighed the signal.

Recovery at 3.2 kPa was close to zero for all binders, ranging from −4.4% to 5.8%, and every point lies far below the M332 elastic-response curve (Figure 3b). The slightly negative values, which are largest for the softest binder, correspond to strain that continues to increase after unloading and are consistent with a residual stress of the order of 1 Pa during the recovery phase; they are reported as being unaltered. The near-zero recovery at 3.2 kPa is material behavior, consistent with the purely viscous response identified by the fractional model, with α ≈ 1 (Section 3.3), and with the behavior of plastomer-modified binders [26,28]. The negative sign, however, is a measurement effect: strain cannot continue to increase after unloading unless a small stress remains, and the magnitude of the negative values increases with the compliance of the binder, as expected for a residual stress of about 1 Pa, about 0.03% of the applied stress, which is within the typical zero-torque offset of a stress-controlled rheometer. Consistent with this explanation, the more compliant unaged base bitumen gave larger negative values, from −3.2% at 64 °C to −12.4% at 82 °C (Table S9). The recovery at 3.2 kPa should therefore be read as zero within the measurement resolution. At 0.1 kPa, W6H6 showed clear recovery that decreased steadily with temperature, from 34.1% at 64 °C to 22.0%, 12.9% and 5.6% at 70, 76 and 82 °C. This recovery reflects material behavior, as it decreased smoothly with temperature in step with the increase in the fractional order (Section 3.3). W4H8 recovered 4.4% at 70 °C and 18.4% at 76 °C. The stress sensitivity J_nr,diff_ of the blends was 73–130%, above the 75% M332 limit in all conditions except W6H6 at 82 °C (Figure 3c), so the modified binders were markedly stress-sensitive.

Table 4 gives the temperatures up to which the AASHTO M332 limits on J_nr,3.2_ were met; because these limits are defined for RTFOT residue, they are indicative for the PAV residues tested here. The control did not meet the Standard-traffic limit even at 64 °C, where its J_nr,3.2_ was 6.96 kPa^−1^. W4H8 met the Standard, Heavy and Very Heavy limits up to 73.2, 68.1 and 64.3 °C, and W6H6 met the Standard and Heavy limits up to 70.0 and 64.7 °C. These temperatures are far below the >82 °C that the oscillatory |G*|/sinδ values of the modified binders would appear to imply (Figure 2a), a discrepancy that Section 3.4, Section 3.5, Section 3.6, Section 3.7, Section 3.8 and Section 3.9 explain.

### 3.3. Fractional Viscoelastic Modeling of Creep and Recovery

**Figure 4:**
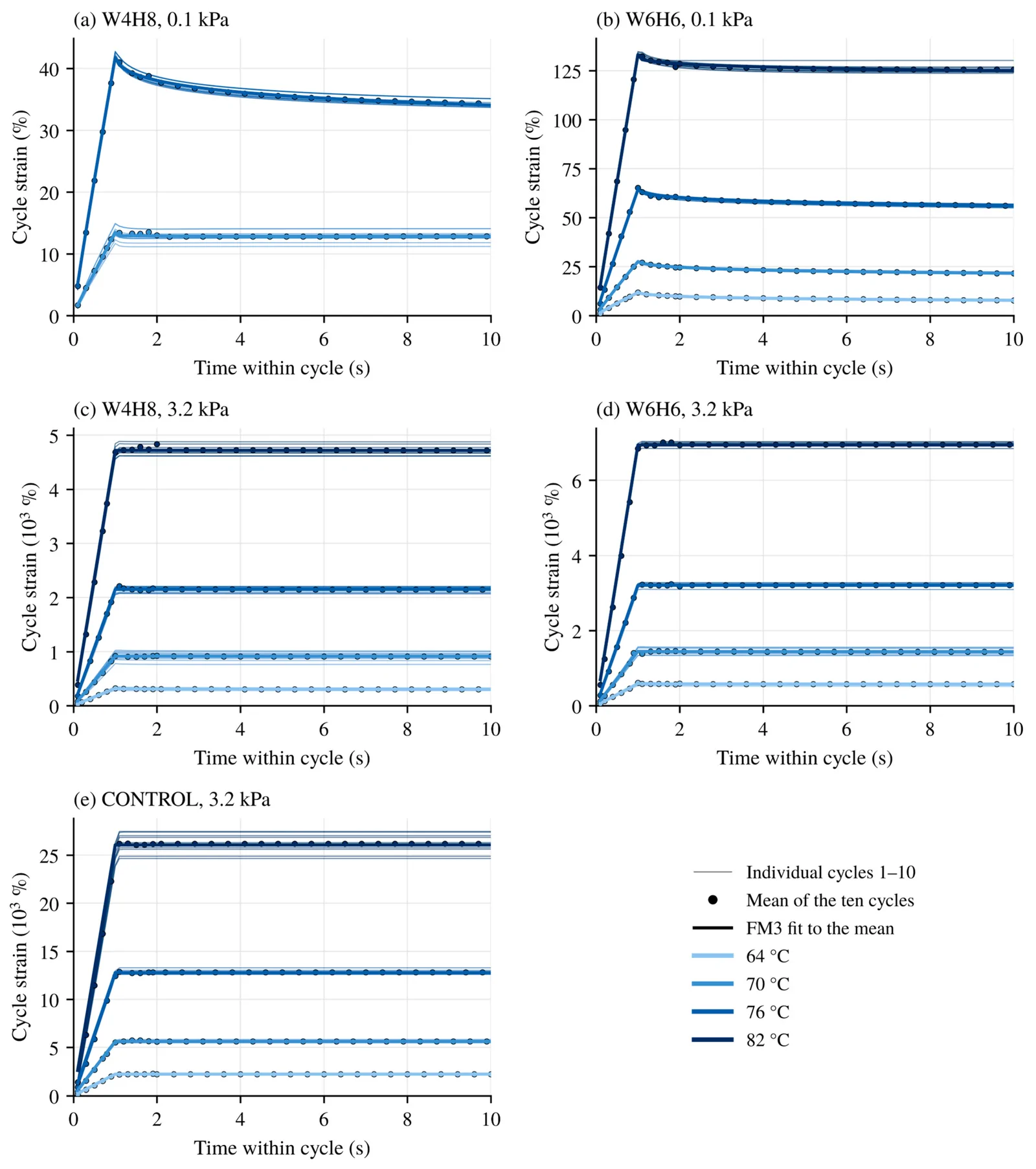
MSCR cycles of the PAV-aged binders: the ten individual cycles, thin lines; their mean, markers, every fourth point shown; and the three-parameter fractional Maxwell fit to the mean, thick lines, for (**a**) W4H8 and (**b**) W6H6 at 0.1 kPa and (**c**) W4H8, (**d**) W6H6 and (**e**) CONTROL at 3.2 kPa. Temperatures shown are those at which each stage could be reported, as explained in Section 3.2.

The three-parameter fractional Maxwell model reproduced all 18 mean cycles with R^2^ = 0.989–0.999 and NRMSE ≤ 2.3% (Table 5, Figure 4), and the model-derived J_nr_ agreed with the cycle-averaged values within 1%. The lowest R^2^ occurred for the control at 82 °C, where the creep strain exceeds 2 × 10^4^% and the residual-stress drift during recovery (Section 3.2) is largest. Figure 4 shows the ten individual cycles together with their mean, and Figure S3 gives the complete strain–time record of each test. The model was fitted to the mean cycle because the ten cycles at each stress differed little, as shown in Section 3.10; the growth of the creep strain from cycle to cycle is analyzed separately in Section 3.8.

**Figure 5:**
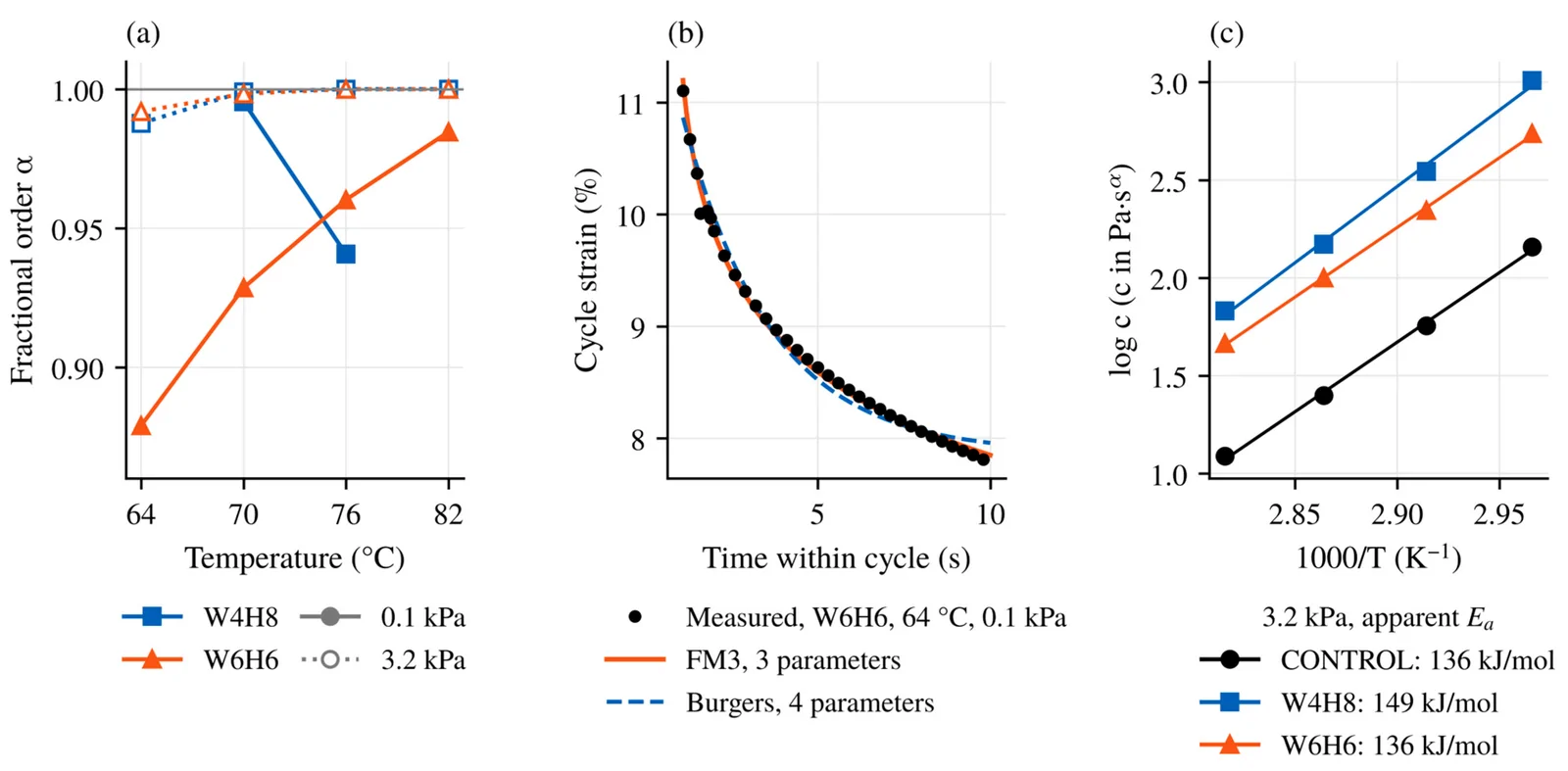
(**a**) Fractional order α from the three-parameter fractional Maxwell fits of the modified binders at 0.1 and 3.2 kPa. (**b**) Recovery phase of W6H6 (64 °C, 0.1 kPa): mean of ten cycles with the fractional Maxwell (three parameters) and Burgers (four parameters) fits; ΔAICc = 146 in favor of the fractional model. (**c**) Arrhenius plot of the springpot constant c from the 3.2 kPa fits, with apparent activation energies.

Two regimes emerged (Figure 5a). At 3.2 kPa, every binder was described by α between 0.988 and 1.000, and for most runs the spring modulus G reached its upper bound, so that the fit reduced to a single dashpot. Over the 1–10 s window of the test, the response at 3.2 kPa is therefore viscous flow with negligible recoverable deformation, and the additional parameters of FM4 and the Burgers model brought no substantial improvement: |ΔAICc| was below 10 in every case and mostly −2.1, the penalty for an unused parameter.

At 0.1 kPa the blends retained some delayed elasticity. The fractional order of W6H6 increased systematically with temperature, from 0.879 at 64 °C to 0.929, 0.960 and 0.985 at 70, 76 and 82 °C, mirroring the decrease of %R_0.1_. Its delayed recovery was clearly better described by the power-law decay of the springpot than by the single exponential of the Burgers model: at 64–76 °C the three-parameter FM3 had a lower AICc than the four-parameter Burgers model by 53–146 units (Table 5, Figure 5b). FM4 improved on FM3 at 76 and 82 °C, by 33 and 94 AICc units, indicating a second, faster power-law process once the recovery became small, and only at 82 °C did the Burgers model outperform FM3. W4H8 gave α = 0.995 at 70 °C and 0.941 at 76 °C, in line with its recovery at these temperatures; FM3 was again preferred to the Burgers model at 76 °C, by 40 units. The creep-fitted α values of 0.879–1.000 are much larger than the oscillatory values α_sp_ = δ/90° of the same blends, which ranged from 0.09 to 0.58 (Figure 2b), a first indication that the oscillatory and creep tests probe different states of the material.

The springpot constant c of the 3.2 kPa fits, which for α ≈ 1 is effectively a viscosity, followed an Arrhenius dependence on temperature for every binder, with R^2^ ≥ 0.997 (Figure 5c and Section 3.9). Bootstrap resampling of the cycles changed α by at most ±0.004 (Section 3.10), so the trend of α with temperature for W6H6 at 0.1 kPa, from 0.879 to 0.985, is far larger than its uncertainty.

**Figure 6:**
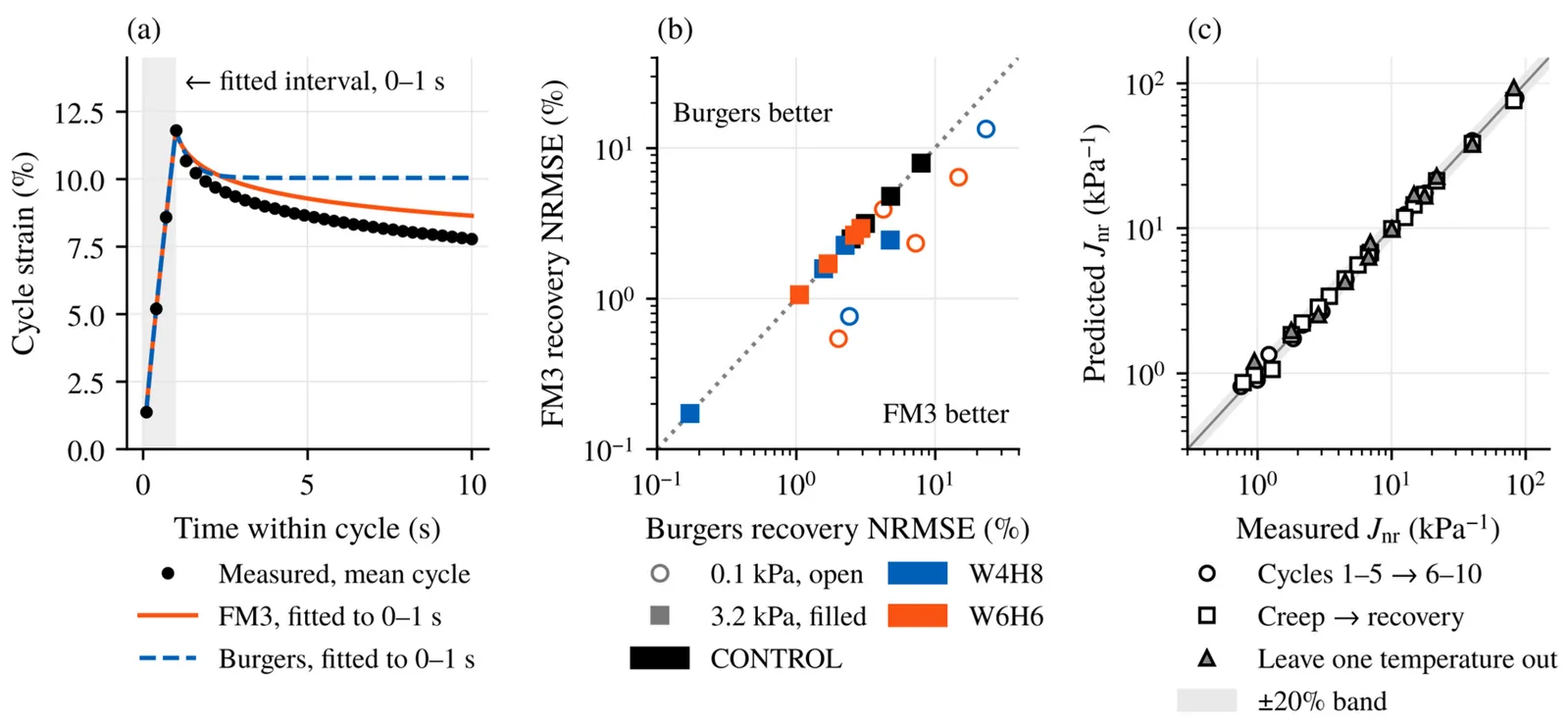
Independent validation of the three-parameter fractional Maxwell model. (**a**) Mean cycle of W6H6 at 64 °C and 0.1 kPa with the FM3 and Burgers models fitted to the creep phase only, 0–1 s, and extended to the recovery phase. (**b**) Error of the predicted recovery, FM3 against Burgers, for all stages; points below the 1:1 line favor FM3. (**c**) Predicted against measured J_nr_ in the cycle split, the phase split and the leave-one-temperature-out test; the shaded band marks ±20%.

The three validation tests confirm that the model predicts data to which it was not fitted (Figure 6; Table S7). In the cycle split, J_nr_ of cycles 6–10 was predicted within 3% in 12 of the 18 stages. In the other six, the error of 6–11% matched to within about 1 percentage point to that of simply reusing cycles 1–5, which reflects the cycle-to-cycle strain accumulation of Section 3.8 rather than a deficiency of the model. In the phase split, the model fitted to the creep phase alone predicted the recovery curve with an NRMSE of at most 8% in 17 of the 18 stages, and in the seven stages with measurable recovery its error was 1.1–3.7 times lower than that of the Burgers model fitted in the same way (Figure 6a,b). For W6H6 at 64 °C and 0.1 kPa, for example, the recovery predicted from the creep phase alone was 27.0%, against a measured 34.1% and a Burgers prediction of 14.9%. In the temperature split, J_nr,3.2_ was predicted within 2–11% at the interpolated temperatures of 70 and 76 °C and within 5–28% at the extrapolated temperatures of 64 and 82 °C (Figure 6c).

The parameters were also well determined (Table S8). The standard errors of log c and α were at most 0.002 in every fit, much smaller than the changes of α with temperature discussed above. The spring modulus G was identifiable in only 7 of the 18 fits; in the others it reached its upper bound, and J_nr_ was insensitive to it in all fits. J_nr_ was inversely proportional to c and very sensitive to α: a 1% change in α changed J_nr_ by about 2.8%, and a change of 0.01 in α changed %R by 2–3 percentage points. Because c and α were strongly correlated, with correlation coefficients of 0.87–0.94, the two parameters should be interpreted together rather than separately.

### 3.4. Departure from Linear Viscoelasticity (Λ Ratio)

**Figure 7:**
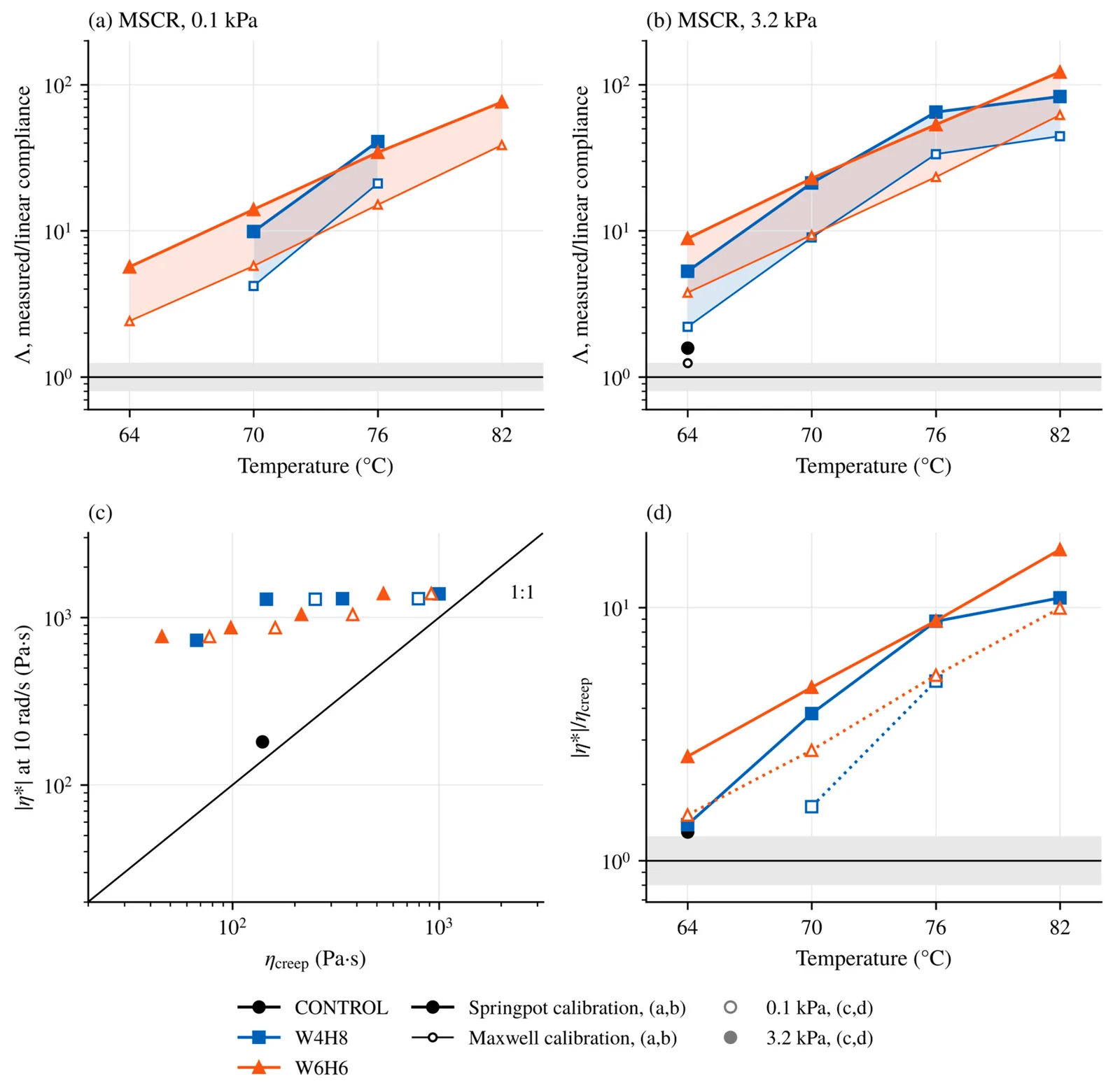
(**a**,**b**) Ratio Λ of the measured 1 s creep compliance to the linear prediction from the oscillatory data at (**a**) 0.1 kPa and (**b**) 3.2 kPa. Thick lines with filled symbols: springpot calibration (upper bound of Λ); thin lines with open symbols: Maxwell calibration (lower bound); shaded areas span the two. (**c**) Complex viscosity at 10 rad/s against the apparent creep viscosity from the steady part of the MSCR creep phase (filled symbols 3.2 kPa, open symbols 0.1 kPa); (**d**) ratio |η*|/η_creep_ versus temperature (solid lines 3.2 kPa, dotted lines 0.1 kPa). The gray bands mark ratios of 0.8–1.25.

Table 6 and Figure 7a,b compare the measured 1 s creep compliance with the linear predictions from the oscillatory data. For the control at 64 °C, the temperature at which both measurements are available, the measured compliance at 3.2 kPa exceeded the linear prediction by a factor of only 1.25 with the Maxwell calibration and 1.58 with the springpot calibration, although the MSCR creep strain reached 2210%. This agrees with the good linear predictions reported for unmodified binders [37] and confirms that the single-point prediction is reasonable for a homogeneous binder; the modest excess is consistent with the mild shear thinning expected at a creep shear rate of 23 s^−1^ (Section 3.5). The same check on the base bitumen before aging, tested with the same protocols, gave Λ = 0.91–1.07 at 0.1 kPa and 1.02–1.35 at 3.2 kPa over the whole range of 64–82 °C (Table S9), which confirms that the single-point prediction holds for a linear binder at all test temperatures and at both stress levels.

The modified binders departed from linear behavior by one to two orders of magnitude. At 0.1 kPa, Λ of W6H6 increased from 5.7 at 64 °C to 76 at 82 °C with the springpot calibration, or from 2.4 to 39 with the Maxwell calibration; W4H8 gave Λ = 9.9 and 41 at 70 and 76 °C. At 3.2 kPa the departure was larger still: for W6H6 Λ rose from 8.9 to 122 with the springpot calibration and from 3.8 to 62 with the Maxwell calibration between 64 and 82 °C, and for W4H8 from 5.3 to 83 and from 2.2 to 44. Even at 64 °C, the lower bound of the blends, given by the Maxwell calibration, was 2.2–3.8 and exceeded the control value of 1.25, and at 82 °C it reached 44–62. Λ increased monotonically with temperature for both blends at both stresses, and at each temperature it was larger at 3.2 kPa than at 0.1 kPa.

These results are consistent with the observations in Section 3.1, Section 3.2 and Section 3.3. In the oscillatory test, the blends respond as a stiff, increasingly elastic solid, with δ down to 8°, and a linear prediction from that state would imply 1 s creep strains of only a few percent at 0.1 kPa and of the order of 10–100% at 3.2 kPa. In MSCR the same binders reached 12–133% at 0.1 kPa and 320–6800% at 3.2 kPa and flowed almost as viscous liquids, with α approaching 1. The load-bearing structure that controls the oscillatory response does not survive these strains.

### 3.5. Creep Viscosity and the Cox–Merz Rule

The viscosity comparison of Equation (12), shown in Table 6 and Figure 7c,d, leads to the same conclusion without any constitutive assumption. For the control at 64 °C, |η*| at 10 rad/s exceeded η_creep_ by a factor of 1.30, or 1.17 when evaluated at the creep shear rate of 23 s^−1^, which is close to the Cox–Merz expectation for a nearly Newtonian binder. For the blends, |η*| exceeded η_creep_ by factors of 1.5–10.0 at 0.1 kPa and 1.4–16.9 at 3.2 kPa, increasing with temperature at both stresses (Figure 7d). Only W4H8 at 64 °C and 3.2 kPa, with a ratio of 1.39, was close to the control value; its rate-matched ratio of 2.8 was not. Because the blends behave as solids in the oscillatory test, with α_sp_ = 0.09–0.41 at 64–82 °C, the rate-matched comparison is very sensitive to the extrapolation: it gives 2.6–4.4 at 3.2 kPa, where creep rates of 3–70 s^−1^ were close to 10 s^−1^, and 22–93 at 0.1 kPa, where they were 0.1–1.3 s^−1^. In every case the modified binders violate the Cox–Merz rule, which confirms that the excess creep identified by Λ is not an artifact of the springpot or Maxwell calibration.

### 3.6. Stress-Softening of the Creep Viscosity

**Figure 8:**
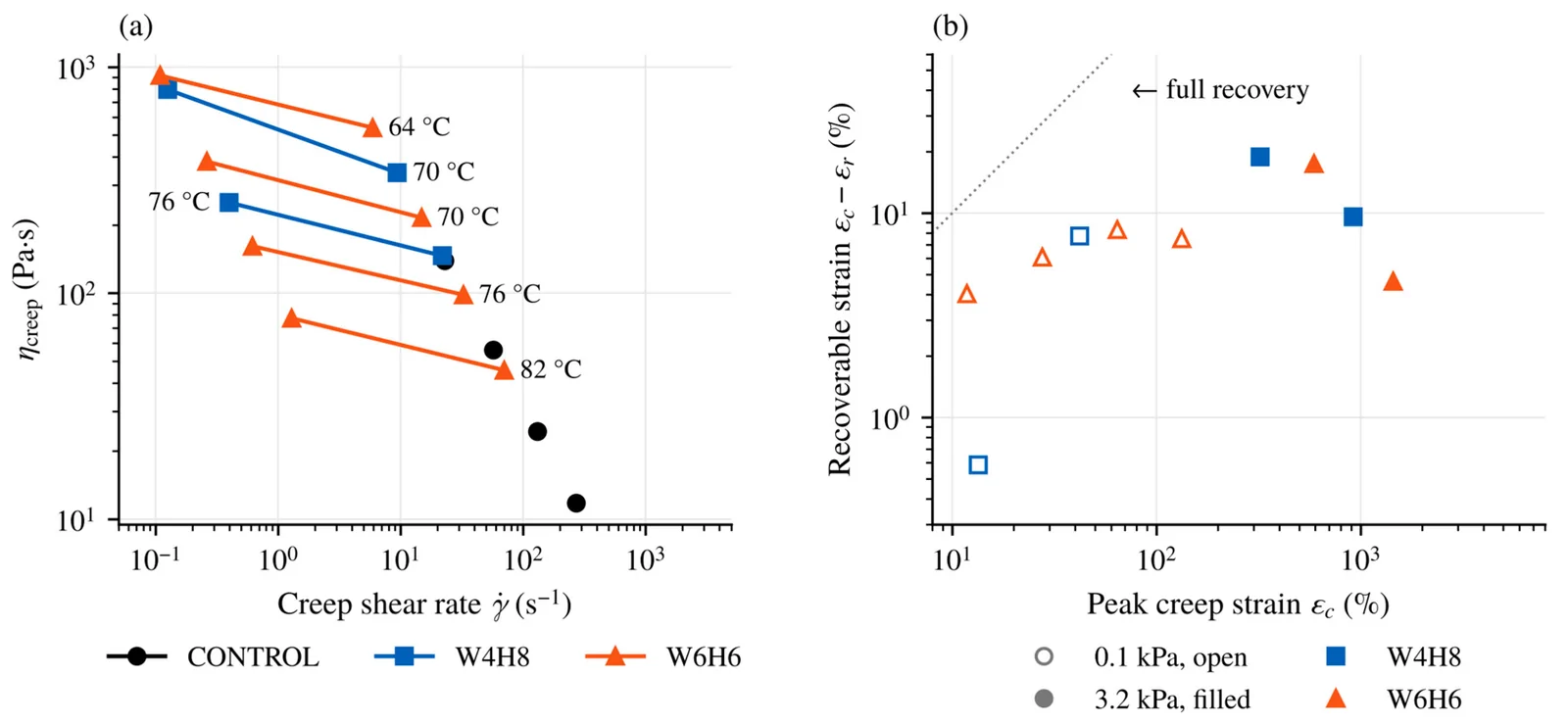
Indicators of structural breakdown obtained from the MSCR data alone: (**a**) apparent creep viscosity against creep shear rate at 0.1 and 3.2 kPa, with lines joining the two stress levels of each test and n from Equation (13); (**b**) recoverable strain against peak creep strain for the MSCR stages of the blends with positive recoverable strain, with open symbols for 0.1 kPa and filled symbols for 3.2 kPa. The values for all stages are given in Supplementary Table S5.

Figure 8a shows the creep viscosity of the blends at the two stress levels, and the numerical values are given in Table S6. For both blends η_creep_ fell by a factor of 1.6–2.3 when the stress rose from 0.1 to 3.2 kPa, while the creep shear rate rose 52–75-fold, giving a flow index n of 0.80–0.87 (Equation (13)). For W6H6 n was almost independent of temperature, at 0.86–0.87. The blends are therefore stress-softening: the larger the deformation, the lower their resistance to flow, which is the MSCR counterpart of the J_nr,diff_ values above 75% (Section 3.2) and of the departure from the Cox–Merz rule (Section 3.5). Because the 0.1 kPa stage of the control could not be used, as noted in Section 3.2, its flow index could not be obtained in the same way; at 3.2 kPa its creep viscosity decreased from 139 to 12 Pa·s between 64 and 82 °C (Figure 8a).

### 3.7. Elastic Strain Capacity

Figure 8b relates the recoverable strain of each MSCR stage to its peak creep strain. If the blends stored elastic strain in proportion to the imposed deformation, as a linear material does, the points would follow a line parallel to the full-recovery diagonal. Instead, the recoverable strain stayed within about 4–19% while the peak strain increased from 12% to about 1440%, i.e., by more than two orders of magnitude (Figure 8b). For W6H6 at 0.1 kPa the recoverable strain rose only from 4.0% to 8.3% between 64 and 76 °C and was 7.5% at 82 °C, while the peak strain increased from 12% to 133%; at 3.2 kPa and 64 °C, both blends recovered 17–19% of strain out of a peak of 320–590%. The structure therefore has a limited elastic strain capacity, of the order of 10% shear strain, beyond which additional deformation is not stored but dissipated as flow. W4H8 at 70 °C and 0.1 kPa, with 0.6%, was the only stage well below this range. At 3.2 kPa and 76–82 °C the recoverable strain was negative (Section 3.2), so no capacity can be read from these stages.

### 3.8. Strain Accumulation over Successive Cycles

**Figure 9:**
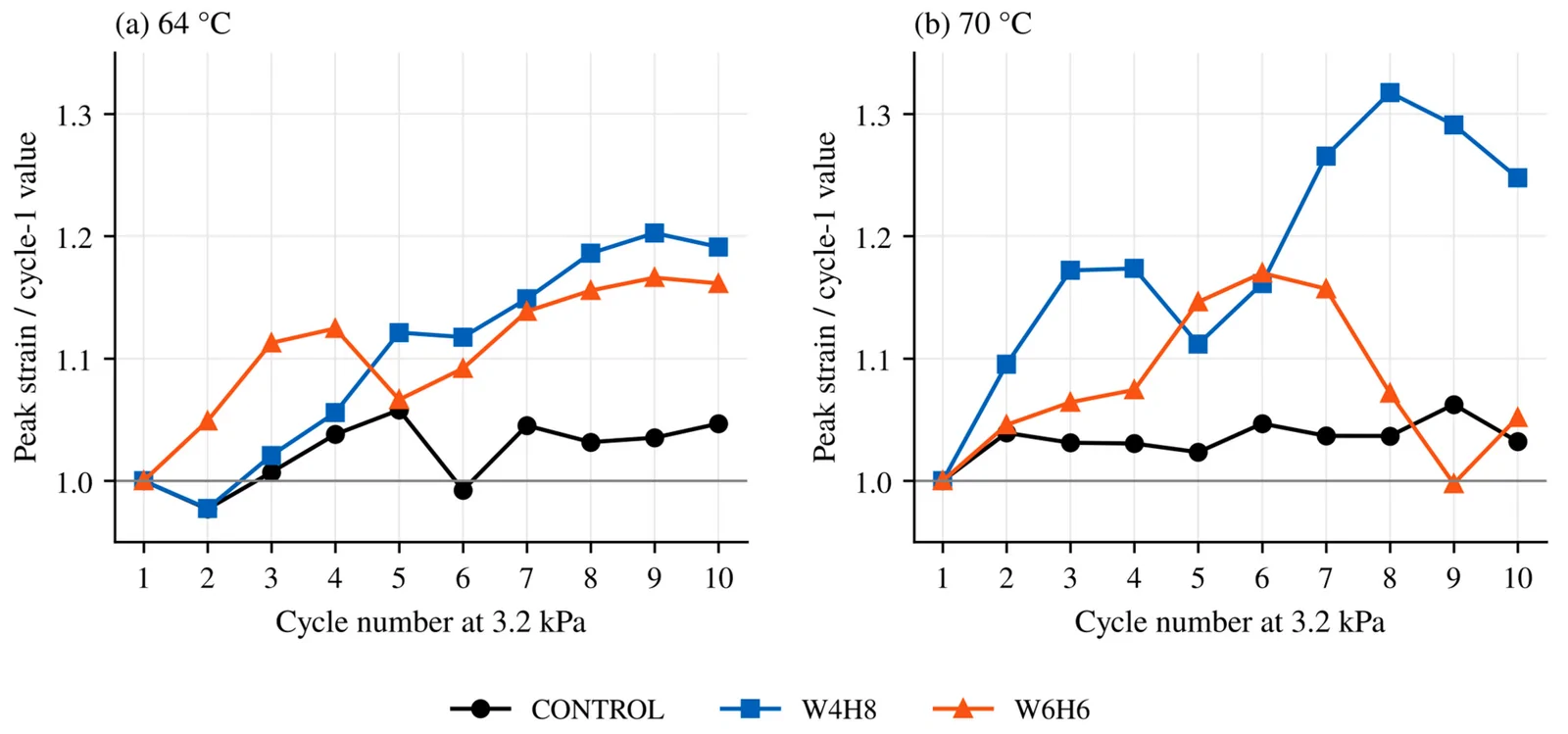
Peak creep strain of each 3.2 kPa cycle normalized by that of the first cycle at (**a**) 64 °C and (**b**) 70 °C.

Further evidence of progressive structural breakdown comes from the evolution of the peak creep strain over the ten cycles at 3.2 kPa (Figure 9). For the control the peak strain of cycle 10 was within 5% of that of cycle 1 at 64 and 70 °C, with ratios of 1.047 and 1.032, and the between-cycle variation was 1.5–2.6%. For the blends the peak strain grew from cycle to cycle at the lower temperatures: by 19% for W4H8 and 16% for W6H6 at 64 °C and by 25% for W4H8 at 70 °C, with mean drifts of 1.6–2.8% per cycle. At 76 and 82 °C the ratio of the cycle-10 to the cycle-1 peak strain of the blends was 0.97–1.05, i.e., no systematic accumulation remained. Progressive softening is therefore seen where the structure detected in oscillation is most developed relative to the matrix, that is, at the lower temperatures, and disappears at the temperatures where Λ is largest and the response from the first cycle onwards is already dominated by flow.

### 3.9. Temperature Dependence: Thermorheological Complexity and Activation Energy

**Figure 10:**
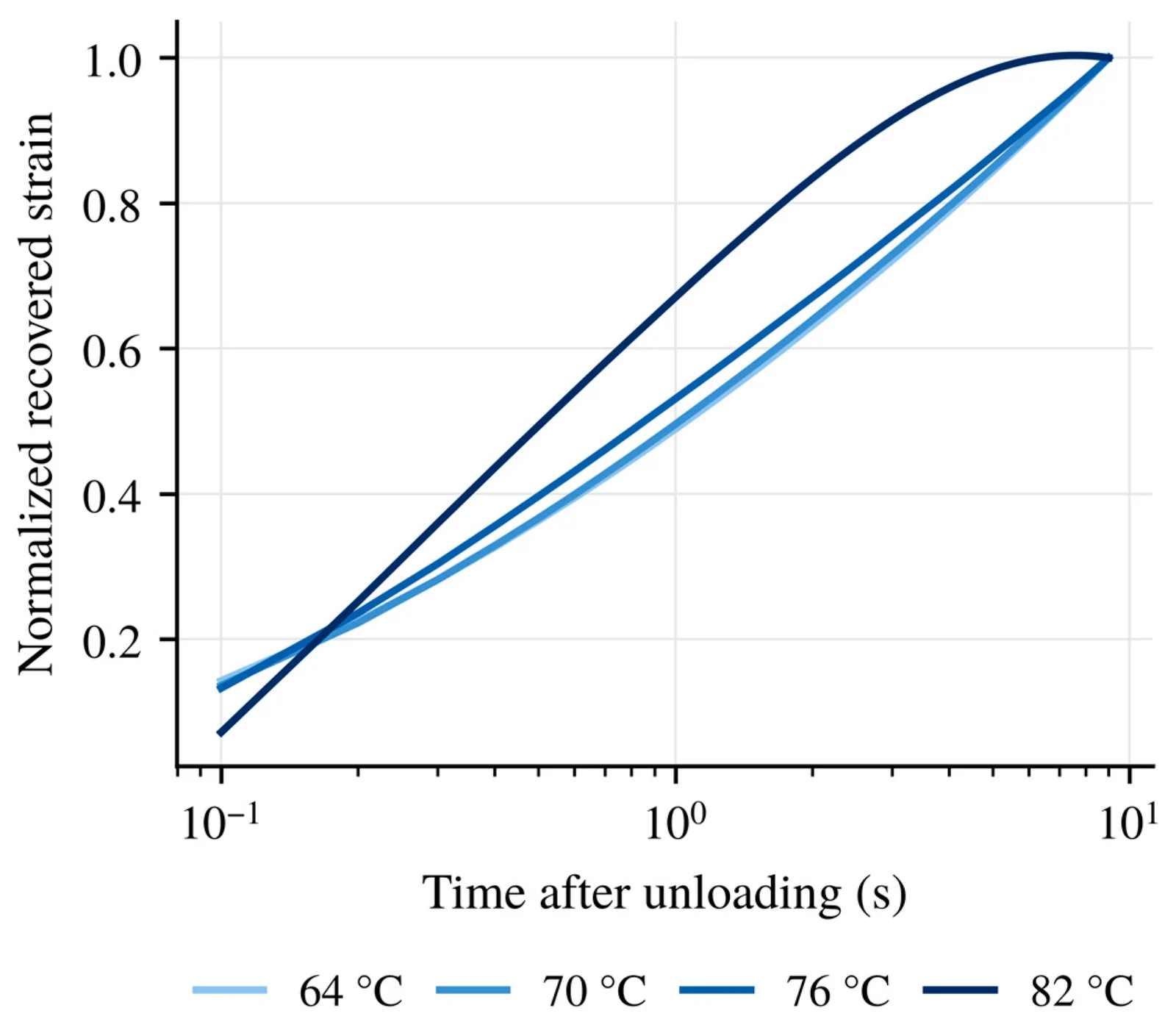
Recovered strain of W6H6 at 0.1 kPa, normalized by its value 9 s after unloading, against time after unloading, at 64–82 °C.

Figure 10 tests whether the recovery of W6H6 at 0.1 kPa obeys time–temperature superposition. Normalized by the recovered strain at 9 s, the recovery curves at 64 and 70 °C were almost identical, and the curve at 76 °C differed from them by an RMS of 0.015; the best horizontal shift onto the 64 °C curve was log a_T_ = 0.01 at 70 °C and 0.04 at 76 °C, so the recovery time scale changed by less than 10%. Over the same range, the creep viscosity of W6H6 at 0.1 kPa decreased 5.7-fold (Table S6) and J_nr,3.2_ increased 5.6-fold (Figure 3a). Viscous flow and delayed recovery therefore have very different temperature dependences: flow follows the softening matrix, whereas the recovery kinetics, like the nearly constant G′ of Section 3.1, appear to be governed by the HDPE-rich structure. At 82 °C the recovery became faster, with log a_T_ = 0.34, and changed shape, with an RMS mismatch of 0.036, in line with the larger fractional order of 0.985 at this temperature and with the improvement of FM4 over FM3 (Section 3.3). No single shift factor can superpose both the creep and the recovery of the blends, which are therefore thermorheologically complex; the control, whose response at 3.2 kPa is purely viscous with α = 1.000, shows no such conflict.

Table 7 compares the apparent activation energies. For the control, the activation energy of the oscillatory modulus over 52–64 °C, 129 kJ/mol, was similar to that of the creep quantities over 64–82 °C, 136 ± 4 kJ/mol from both J_nr,3.2_ and c, as expected for a binder whose oscillatory and creep responses are governed by the same relaxation processes.

The blends behaved differently. Over 52–64 °C, where the bitumen matrix still contributes substantially, their oscillatory activation energy of 84–85 kJ/mol was already lower than that of the control. Over 64–82 °C it fell to 32–33 kJ/mol, and for W4H8 the Arrhenius regression was poor, with R^2^ = 0.68, because |G*| hardly changed between 64 and 76 °C. In creep over the same range, however, their activation energies were 138 ± 3 kJ/mol for W6H6 and 151 ± 7 kJ/mol for W4H8, equal to or slightly above that of the control. The creep response of the blends was therefore 4.2–4.8 times more temperature-sensitive than their oscillatory response. The temperature insensitivity seen in oscillation, a property usually associated with a durable elastic network, does not carry over to creep: at MSCR strains the blends soften with temperature at least as quickly as the base binder, and their advantage lies in a lower absolute J_nr_ rather than in a flatter temperature dependence. The activation energy from the springpot constant c agreed with that from J_nr,3.2_ within 2 kJ/mol for every binder, as expected when α ≈ 1.

### 3.10. Uncertainty of the Main Results

Table 8 gives bootstrap 95% confidence intervals. The intervals on J_nr_ were within ±5% of the mean for all conditions, and those on Λ within ±5%. The ratio of J_nr,3.2_ of W4H8 to that of W6H6 was 0.53 at 64 °C, with a 95% confidence interval of 0.51–0.55, and 0.63–0.68 at 70–82 °C, with intervals no wider than ±0.04, so the difference between the blends is resolved at every temperature. The reductions relative to the control were 5.5–7.3 for W4H8 and 3.8–4.0 for W6H6, with confidence intervals of 5.4–7.6 and 3.7–4.1. Recovery at 0.1 kPa was resolved to within about ±1 percentage point, whereas %R_3.2_ of the blends remained within ±2% at 70 °C and above, with intervals of about ±0.7 percentage points, i.e., negligible in practical terms. Because the confidence intervals reflect only cycle-to-cycle variation within one specimen, they demonstrate that the reported contrasts are not caused by scatter between cycles. Each temperature was, however, tested on a separate specimen, and the J_nr,3.2_ values of each binder deviated from their Arrhenius fit by at most 4% for the control, 4% for W6H6 and 9% for W4H8. These deviations bound the combined effect of specimen-to-specimen variability and model error at about 10%, far less than the 3.8–7.3-fold reduction relative to the control, the 2–122-fold excess creep measured by Λ and the 32–47% difference between the two blends. The same conclusion is reached from independent indicators, namely Λ, the Cox–Merz ratio, the flow index, the recoverable strain, the cycle-to-cycle strain accumulation and the activation energies, at all temperatures and both stress levels, and the combination of a large J_nr_ reduction with near-zero recovery agrees with that reported for polyethylene-modified binders in a multi-laboratory round robin [12]. Neither check, however, replaces replicate specimens.

### 3.11. Indirect Evidence of the HDPE-Rich Structure

**Figure 11:**
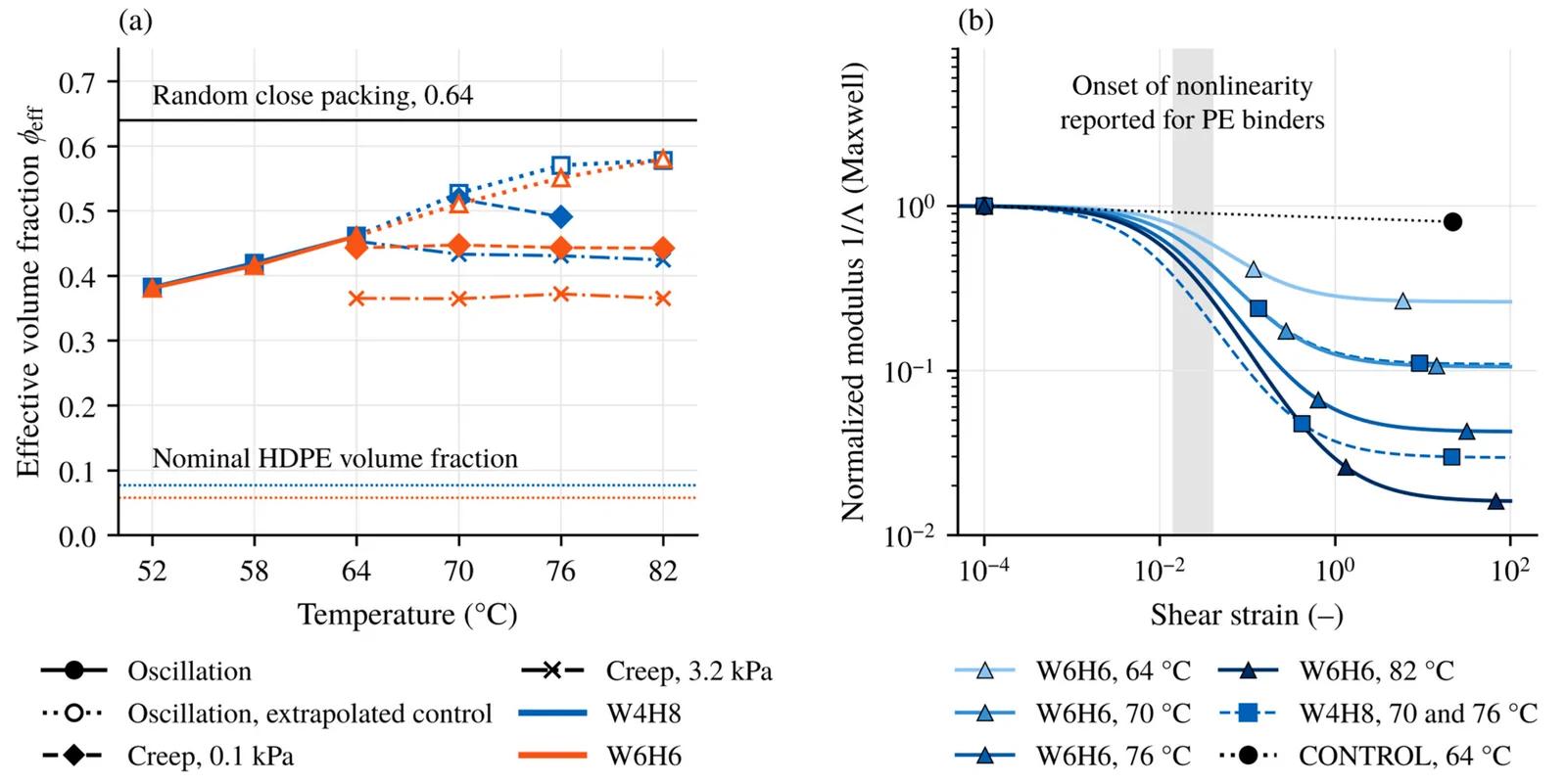
Indirect evidence of the HDPE-rich structure. (**a**) Effective volume fraction of the HDPE-rich phase from the Krieger–Dougherty model, in oscillation and in creep; open symbols use the extrapolated modulus of the control above 64 °C, and horizontal lines mark random close packing and the nominal HDPE volume fractions. (**b**) Normalized modulus 1/Λ, Maxwell calibration, against shear strain, with Kraus fits; the shaded band marks the onset of nonlinearity reported for polyethylene-modified binders [33].

Figure 11a shows the effective volume fraction of the HDPE-rich phase. Rigid HDPE particles at their nominal volume fractions of 0.058 and 0.077 would raise the modulus by only 16–23%, whereas in oscillation the blends were 4.3–7.7 times stiffer than the control at 52–64 °C and about 13–44 times stiffer at 70–82 °C, where the modulus of the control was extrapolated. The corresponding φ_eff_ was 0.38–0.46 at 52–64 °C and increased to 0.51–0.58 at 70–82 °C, approaching random close packing. The HDPE-rich phase therefore acts as if it occupied about 5–10 times its nominal volume, which indicates polymer domains swollen by the light fractions of the bitumen and the WEO; at the higher temperatures these domains are close enough to touch. In creep the picture is different: φ_eff_ was 0.37–0.45 at 3.2 kPa and 0.44–0.52 at 0.1 kPa and hardly changed with temperature, as expected for swollen domains that are separated by flow and act as a dispersed filler rather than as a connected structure.

Figure 11b shows 1/Λ against strain with the Kraus fits. For the blends, the critical strain was 0.4–3.1%, depending on the binder, the temperature and the calibration; it decreased with increasing temperature and was lower with the springpot calibration than with the Maxwell calibration. These values agree with the onset of nonlinearity at 1.4–4.1% strain reported in amplitude sweeps of polyethylene-modified binders [33], and they are 40–300 times larger than the oscillatory strain amplitude of 0.01%. With the Maxwell calibration, the limiting modulus g_∞_ of W6H6 fell from 0.26 at 64 °C to 0.016 at 82 °C, so the fraction of the small-strain modulus retained at MSCR strains decreased as the matrix softened. For the control, 1/Λ remained at 0.8 at a creep strain of more than 2000%.

## 4. Discussion

### 4.1. Proposed Interpretation: A Strain-Fragile, HDPE-Rich Structure

**Figure 12:**
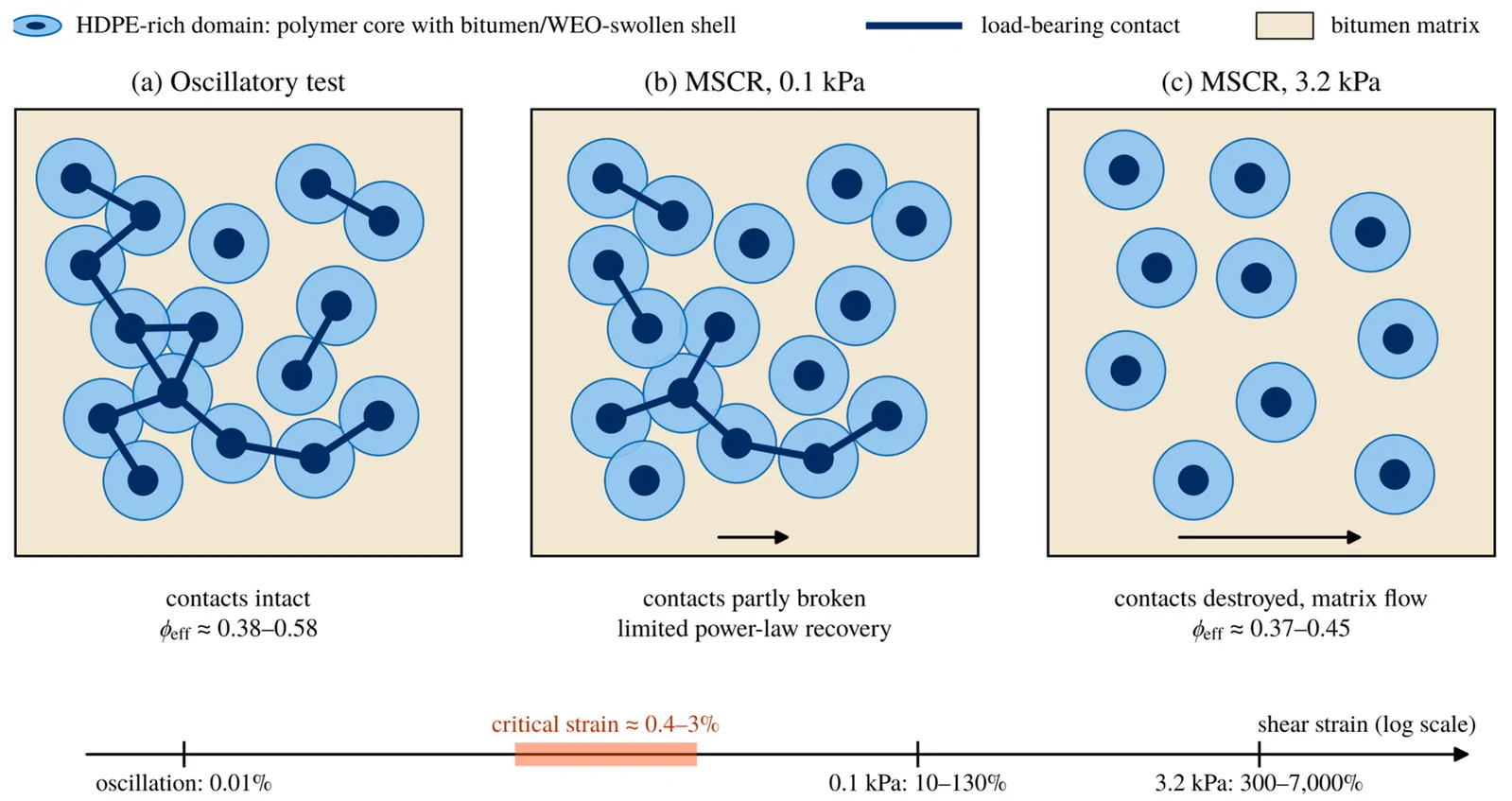
Schematic interpretation, not to scale. HDPE-rich domains consist of a polymer core with a shell swollen by bitumen and WEO. (**a**) In the oscillatory test, contacts between the domains form a load-bearing path that dominates the high-temperature response, with an effective volume fraction of 0.38–0.58. (**b**) At 0.1 kPa in MSCR, with strains of 10–130%, part of this path breaks and the response becomes predominantly viscous with limited power-law recovery. (**c**) At 3.2 kPa, with strains of 300–7000%, the path is destroyed, the domains act as a dispersed filler with an effective volume fraction of 0.37–0.45, and flow of the WEO-plasticized matrix governs. The strain axis marks the critical strain of about 0.4–3% estimated in Section 3.11.

The results describe binders whose apparent stiffness depends on strain amplitude by one to two orders of magnitude. In the oscillatory test, the aged HDPE/WEO binders behave increasingly like solids as the temperature rises: the bitumen matrix softens, but a stiff structure with G′ ≈ 10^4^ Pa remains and dominates the response, driving δ down to 8–10° at 82 °C. Under the strains of the MSCR test, this structure carries very little load: the binders flow with α close to 1, recover little, creep 2–122 times more than the oscillatory data would predict, violate the Cox–Merz rule, and, at the lower temperatures, soften further from cycle to cycle. The control, by contrast, is predicted within a factor of 1.3–1.6 even at a creep strain of 2210%. The MSCR data alone point the same way: the blends are stress-softening, with n = 0.80–0.87, store only about 4–19% recoverable strain regardless of the imposed deformation, and recover on a time scale that does not follow the temperature shift of viscous flow. The difference between the two classes of binder is therefore not a matter of measurement scatter or model choice, but of a nonlinearity introduced by the modifier (Figure 12). The indirect structural analysis is consistent with this interpretation: the HDPE-rich phase acts as if it occupied 5–10 times its nominal volume, its effective volume fraction approaches random close packing in oscillation but not in creep, and the structure begins to break down at strains of about 0.4–3% (Section 3.11).

This behavior closely resembles the Payne effect in filled elastomers, in which a network of filler–filler contacts gives a high storage modulus at low strain amplitudes and is progressively broken as the amplitude increases [36,61]. In HDPE-modified bitumen, the dispersed polyethylene is semicrystalline and its crystalline domains melt only far above the test temperatures [65,66]; at 52–82 °C the polymer phase therefore behaves as a stiff, deformable dispersed phase in a matrix that becomes very soft as the temperature increases. Where these domains touch or are bridged by thin polymer-rich layers, they can form a load-bearing path. The decreasing phase angle with temperature is then explained naturally: the matrix contribution to G″ falls rapidly with temperature while the elastic contribution of the HDPE-rich path changes little, so the path increasingly dominates. The increase in Λ with temperature follows from the same picture, because the strains imposed in MSCR at a given stress grow as the matrix softens, and the path is broken more completely. The description of crystalline polyethylene domains as reinforcement in the binder [67] and the sensitivity of polyolefin-modified binder rheology to processing conditions [30,65] are consistent with this interpretation.

An alternative explanation must be considered. If individual HDPE particles or flakes were comparable in size to the 1 mm gap, contacts with both plates could produce a similar oscillatory signature without a binder-scale network. Such jamming would normally be accompanied by large normal forces and an erratic, nearly temperature-independent modulus. For the aged blends the normal force of 0.21–0.30 N was of the same order as that of the control, the response varied smoothly with temperature, the two blends with different HDPE contents gave similar plateau moduli, and the MSCR behavior was consistent between stress levels and across cycles. In addition, the binders were blended at 160 °C, above the melting range of HDPE, under high shear at 4000 rpm for 60 min, which favors a fine dispersion that recrystallizes on cooling rather than intact flakes, although the size of the dispersed HDPE domains after blending was not measured. The effective volume fractions of Section 3.11 also imply that the HDPE acts through swollen domains distributed throughout the binder, which is consistent with a binder-scale structure rather than with a few particles bridging the gap. These observations argue against gap-scale jamming, but they do not prove the existence of a binder-scale network. Measurements at different gaps, amplitude sweeps and microscopy of the polymer phase would distinguish the two explanations directly.

This interpretation should be regarded as a hypothesis consistent with the rheological data rather than as an established microstructure. The effective volume fraction assumes rigid spherical inclusions, the critical strain rests on three strain levels and on the assumption that the oscillatory point is linear, and no morphological observation was made. Other mechanisms could contribute to the same rheological signature, including changes in the crystallinity of the HDPE with temperature, partial phase separation promoted by the WEO, and the particle bridging discussed above. As recent reviews on engineering future binders emphasize, the link between chemistry, structure and rheology remains the least understood aspect of modified binders and requires rheology to be combined with microscopy, spectroscopy and chemical analysis [68].

### 4.2. Consequences for the Characterization and Grading of Plastomer-Modified Binders

The first consequence concerns how stiffness is measured. In the oscillatory test, the blends have |G*|/sinδ of 23–82 kPa at 64–82 °C, far above the 2.20 kPa threshold, which would suggest a high-temperature grade above 82 °C. Even the indicative MSCR traffic levels place the same binders at only 70–73 °C for Standard traffic (Table 4). The two results are reconciled by the strain fragility quantified by Λ: the oscillatory measurement records the intact HDPE-rich structure, whereas rutting under traffic and the MSCR test involve strains at which that structure has largely broken down. The activation energies make the same point for temperature: a binder that appears almost temperature-insensitive in oscillation, with an activation energy of about 32 kJ/mol, softens in creep at least as quickly as the base binder, with 138–151 kJ/mol against 136 kJ/mol. Both tests therefore agree that the modification improves the resistance to permanent deformation: the control did not meet the Standard-traffic limit even at 64 °C, whereas the blends met it up to 70–73 °C, a gain of at least one to two grades. They disagree on its size, because the oscillatory data suggest a gain of three grades or more, which the creep response does not support. For the binders tested here, grading parameters should therefore be measured under creep loading, or at the specified strain amplitude, and oscillatory stiffness on its own should not be used to quantify the improvement in rutting resistance; whether this applies to plastomer-modified binders in general requires validation on a wider range of binders and on asphalt mixtures.

The second consequence concerns the MSCR parameters themselves. The large J_nr_ reduction combined with near-zero %R_3.2_ and J_nr,diff_ above 75% is typical of plastomeric modification [26,28] and should not be read as a failure of the modification. The present analysis provides a mechanistic reading of these numbers: J_nr,0.1_ is measured while part of the HDPE-rich structure is still intact, J_nr,3.2_ after it has broken, so J_nr,diff_ is in effect a measure of structural fragility rather than of elastomeric quality. The ratio Λ, which requires only one oscillatory point and the standard MSCR result at the same temperature, offers a simple, test-compatible index of this fragility, and the model-free viscosity ratio |η*|/η_creep_ provides an independent check of it. Where oscillatory data are not available, the flow index n from the two MSCR stress levels and the recoverable strain of each stage are simple MSCR-only indicators of the same behavior. Because Λ is close to 1 for a linear binder, values well above 1 flag a modified binder whose oscillatory properties overstate its resistance to large deformations.

Both the oscillatory and the MSCR parameters are binder-level indicators, whereas rutting resistance is ultimately a property of the asphalt mixture. J_nr,3.2_ has been shown to correlate better with mixture rutting than |G*|/sinδ, particularly for modified binders [26,28], which is consistent with the present finding that the large strains of the MSCR test, rather than the small strains of the oscillatory test, reveal how the HDPE-rich structure behaves under load. In a mixture, however, the binder acts as a thin film between aggregate particles, and the strains and stresses it experiences depend on the aggregate skeleton, the binder content and the interaction of the modifier with the aggregate surface; partly dispersed polyethylene may also stiffen the mastic in ways that a binder test does not capture. Mixture studies of polyethylene-modified asphalt have generally reported lower rut depths in wheel-tracking tests [69]. The reduction in J_nr,3.2_ found here therefore suggests an improved rutting resistance, but its size can only be established by mixture tests, which are listed as a direction for future research in Section 6.

### 4.3. Fractional Versus Classical Description of Creep and Recovery

The fractional Maxwell model required only three parameters to describe all cycles, and its fractional order carried physical information: α ≈ 1 identified the purely viscous regime that dominated at 3.2 kPa, while the systematic increase in α with temperature for W6H6 at 0.1 kPa quantified the gradual loss of delayed elasticity. For W6H6 at 64–76 °C and W4H8 at 76 °C the power-law recovery of the springpot outperformed the exponential retardation of the four-parameter Burgers model by a wide margin, so the delayed elasticity of these binders is better described as a broad distribution of retardation times than as a single Kelvin–Voigt element [38,43,55]. Two limitations apply. The 10 Hz sampling gives only ten points in each creep phase, so the instantaneous elastic response and very short retardation times are not resolved; and fitted values at the upper bound of G, 10^9^ Pa, indicate that the spring is not identifiable from the data rather than that it is infinitely stiff.

The validation and sensitivity analyses of Section 3.3 clarify the physical meaning of the three parameters. The fractional order α places the response between an elastic solid, with α = 0, and a viscous liquid, with α = 1, and is commonly interpreted as a measure of the breadth of the distribution of relaxation times; its increase toward 1 with temperature for W6H6 at 0.1 kPa tracks the loss of recovery, and because J_nr_ is about three times more sensitive to α than to c, small changes in α have a noticeable effect on the predicted compliance. The springpot constant c, in Pa·s^α^, is a quasi-property that becomes a viscosity as α approaches 1; its activation energy agreed with that of J_nr,3.2_ within 2 kJ/mol for every binder (Section 3.9), which shows that it describes the same flow process. The spring modulus G represents the instantaneous elastic response, which the 10 Hz sampling does not resolve, and it should not be interpreted where it reached its bound. The strong correlation between c and α means that different combinations of the two can describe a given cycle almost equally well, so binders should be compared through α and c together, or through the J_nr_ and %R that they predict, rather than through either parameter alone. The fractional model is nevertheless phenomenological: its parameters describe the response within the 1–10 s window and the 10 Hz sampling of the MSCR test, and they cannot be linked uniquely to a molecular or microstructural mechanism.

### 4.4. Role of the HDPE:WEO Ratio

W4H8, with an HDPE:WEO ratio of 2:1, was stiffer than W6H6, with a ratio of 1:1, in MSCR at all temperatures and met the M332 limits up to higher temperatures, although both blends contain the same total modifier content. In the oscillatory test the two blends were nearly identical up to 64 °C, with W4H8 slightly more elastic above that. The extra WEO in W6H6 therefore softens the matrix, as expected from its role as a rejuvenator [70,71], and reduces the stiffness at large strain, while the extra HDPE in W4H8 raises it. The recovery at 0.1 kPa did not rank the blends consistently: W6H6 recovered far more at 70 °C, 22.0% against 4.4%, whereas W4H8 recovered more at 76 °C, 18.4% against 12.9%. With W4H8 data at 0.1 kPa at only two temperatures and one specimen per condition, no general difference in recovery between the blends can be established.

## 5. Conclusions

The following conclusions apply to the two HDPE/WEO compositions and the PAV-aged state tested in this study. This study compared the oscillatory and creep–recovery responses of two PAV-aged bitumens modified with recycled HDPE and WEO with those of the unmodified base bitumen. Its main finding is that, for these binders, the two tests do not measure the same state of the material. In oscillation at very small strain, the blends behaved increasingly like elastic solids as the temperature rose, which is consistent with a load-bearing structure formed by the HDPE-rich phase. Under the much larger strains of the MSCR test, this structure contributed little: the blends flowed almost as viscous liquids, recovered little and crept far more than their oscillatory stiffness predicted, whereas the response of the unmodified binder was predicted closely. The modification still reduced the non-recoverable creep compliance several-fold, but this benefit appears to come from a stiffer flowing binder rather than from a durable elastic network.

Three practical implications follow for these binders. First, for the binders tested, the oscillatory parameter |G*|/sinδ overstated the size of the improvement in resistance to permanent deformation, which the MSCR test confirmed at a lower level; for such binders it should not be used on its own to grade them or to compare them with elastomer-modified binders; a creep test at stresses representative of traffic, such as MSCR, gives the more relevant measure. Second, the ratio of HDPE to WEO, and not only the total modifier content, governed the creep response: at the same total content, the blend with more HDPE and less oil had the lower compliance, which suggests keeping the oil content to the minimum needed for blending. Third, the low temperature sensitivity seen in oscillation did not carry over to creep, which was as temperature-sensitive as that of the base bitumen, so claims of improved temperature susceptibility for such binders should be verified under creep loading.

From a methodological point of view, the ratio of the measured to the linearly predicted creep compliance may offer a simple screening index for this kind of strain-dependent behavior, obtained from data already produced in routine DSR and MSCR testing. The fractional order of the three-parameter fractional Maxwell model separates viscous flow from delayed elastic recovery with fewer parameters than the Burgers model. Whether oscillatory stiffness also overstates the rutting resistance of other plastomer-modified binders, at other compositions and aging states and in asphalt mixtures, remains to be validated, as outlined in Section 6.

## 6. Limitations and Future Research

This study has some limitations. One specimen was tested per binder and temperature. The bootstrap intervals therefore describe the variability between the cycles of one specimen, not between independent specimens, and the check based on the four specimens of each binder in Section 3.10 bounds the specimen-to-specimen variability only indirectly. The difference between the two blends, which is much smaller than their difference from the control, is the result that most requires confirmation with replicate specimens. The oscillatory tests used a single frequency and strain amplitude, so the strain at which the HDPE-rich structure breaks down was not measured directly. Some reference data for the aged control, at 70–82 °C in oscillation and at 0.1 kPa in MSCR, could not be used; the unaged base bitumen provides a linear reference under these conditions, but not in the aged state, and 58 °C was not tested. The structural interpretation is inferred from rheology without morphological observation. All results for the blends refer to PAV residues of two compositions prepared from one source of each material.

These limitations, together with the findings, suggest four directions for future research.

The first is to link the rheological response directly to the structure of the HDPE phase. Amplitude sweeps with recovery steps would locate the critical strain and show whether the structure rebuilds after unloading. Fluorescence or electron microscopy, calorimetry and infrared spectroscopy would show the size and connectivity of the HDPE-rich domains, their crystallinity at service temperatures, and the role of the WEO in swelling and dispersing them. Combining these methods on the same binders would test whether the strain-fragile structure proposed here is a general feature of plastomer-modified binders, in line with the call of recent reviews to relate the chemistry, structure and rheology of modified binders [68].

The second concerns testing and grading. The Λ ratio could be evaluated on a larger set of binders, including elastomer-modified and other plastomer-modified binders, to establish threshold values above which oscillatory grading should not be used on its own. Frequency sweeps, master curves and the 58 °C grade would provide a complete linear viscoelastic reference. Replicate specimens from separately prepared batches would allow the precision of Λ and of the fractional parameters to be defined, which is needed before they can be proposed as routine indices.

The third concerns formulation and aging. A wider range of HDPE:WEO ratios and total contents, and recycled materials from different sources, would show how the oil content can be optimized for both workability and creep resistance. Testing the same binders unaged, after RTFOT and after RTFOT followed by PAV would separate the effect of the modifiers from that of aging, allow a formal grading and show how aging changes the HDPE-rich structure. Storage-stability tests would confirm whether the blends remain homogeneous at the temperatures used in practice. Particle-size analysis and calorimetry of the recycled HDPE, and chemical analysis of the WEO, including its saturate, aromatic, resin and asphaltene (SARA) fractions and metal content, would allow the effect of the modifier characteristics to be quantified.

The fourth is to move from the binder to the pavement. Bending beam rheometer and linear amplitude sweep tests would cover low-temperature cracking and fatigue, and wheel-tracking tests on asphalt mixtures would show whether the large difference between the oscillatory and creep responses is reflected in rutting performance. Field sections in hot climates, combined with a life-cycle assessment of the recycled modifiers, would show whether HDPE/WEO binders can deliver a practical and environmentally sound use of these waste materials.

## Figures and Tables

**Table 1:** Basic properties of the base bitumen and requirements of ASTM D946 for the 60–70 penetration grade.

Property	Test Method	Result	ASTM D946 Requirement (60–70)
Penetration at 25 °C, 100 g, 5 s (0.1 mm)	ASTM D5	66	60–70
Softening point, ring and ball (°C)	ASTM D36	48.5	—
Ductility at 25 °C, 5 cm/min (cm)	ASTM D113	110	≥100
Rotational viscosity at 135 °C (mPa·s)	ASTM D4402	309	—
Flash point, Cleveland open cup (°C)	ASTM D92	285	≥232
Specific gravity at 25 °C	ASTM D70	1.040	—

**Table 2:** Characteristics of the recycled modifiers. Values marked as typical are literature values for the material type and were not measured in this study.

Property	Recycled HDPE	Waste Engine Oil	Basis
Source	Post-consumer plastic chairs, recycling sources in Abeokuta	Gasoline-powered passenger cars only, local workshop in Abeokuta	This study
Pretreatment	Cleaned and ground	Filtered through a 100 μm sieve, no chemical treatment	This study
Particle size	Approximately 0.5–2 mm	—	This study
Collection and storage	—	Within 2–3 weeks before use	This study
Flash point, Cleveland open cup (°C)	—	110	This study, ASTM D92, mean of three trials
Fire point (°C)	—	135	This study, ASTM D92, mean of three trials
Density at 25 °C (g/cm^3^)	0.94–0.97	About 0.88	Typical value
Melting range (°C)	About 125–135	—	Typical value

**Table 3:** Composition of the binders (modifier contents by mass of base bitumen). All binders were tested after PAV aging.

Binder	Base Bitumen	WEO (wt%)	HDPE (wt%)	Total Modifier (wt%)	HDPE:WEO
CONTROL	60/70	0	0	0	—
W4H8	60/70	4	8	12	2:1
W6H6	60/70	6	6	12	1:1

**Table 4:** Indicative MSCR traffic levels of the PAV-aged binders (highest temperature at which the AASHTO M332 J_nr,3.2_ limit is met, by log-linear interpolation). The lowest test temperature was 64 °C.

Traffic Level, J_nr,3.2_ Limit (kPa^−1^)	CONTROL	W4H8	W6H6
Standard (S), 4.5	<64 °C	73.2 °C	70.0 °C
Heavy (H), 2.0	<64 °C	68.1 °C	64.7 °C
Very Heavy (V), 1.0	<64 °C	64.3 °C	<64 °C
Extremely Heavy (E), 0.5	<64 °C	<64 °C	<64 °C

**Table 5:** FM3 fits to the mean MSCR cycles (c in Pa·s^α^, G in Pa). ΔAICc = AICc(FM3) − AICc(other model): negative values favor FM3; |ΔAICc| > 10 is strong support. ‘≥9 (bound)’: spring not identifiable (fit reduces to a springpot).

Binder	σ_0_ (kPa)	T (°C)	log c	α	log G	R^2^	NRMSE (%)	ΔAICc vs. FM4	ΔAICc vs. Burgers
CONTROL	3.2	64	2.158	1.000	≥9 (bound)	0.9968	1.25	−2.1	−2.1
CONTROL	3.2	70	1.755	1.000	≥9 (bound)	0.9977	1.09	−2.1	−2.1
CONTROL	3.2	76	1.399	1.000	≥9 (bound)	0.9954	1.44	−2.1	−2.1
CONTROL	3.2	82	1.089	1.000	≥9 (bound)	0.9896	2.27	−2.1	−2.1
W4H8	0.1	70	2.888	0.995	4.34	0.9936	1.61	1.2	2.8
W4H8	0.1	76	2.393	0.941	4.42	0.9990	0.56	56.6	−40.2
W4H8	3.2	64	3.009	0.988	4.89	0.9959	1.37	−1.8	−1.3
W4H8	3.2	70	2.544	0.999	≥9 (bound)	0.9963	1.26	−2.1	0.4
W4H8	3.2	76	2.172	1.000	≥9 (bound)	0.9987	0.72	−2.1	−1.3
W4H8	3.2	82	1.831	1.000	≥9 (bound)	0.9987	0.80	−2.1	−2.1
W6H6	0.1	64	2.951	0.879	≥9 (bound)	0.9978	0.78	−2.1	−145.6
W6H6	0.1	70	2.576	0.929	4.68	0.9986	0.66	6.5	−104.1
W6H6	0.1	76	2.203	0.960	4.24	0.9992	0.59	33.4	−53.4
W6H6	0.1	82	1.885	0.985	3.74	0.9994	0.50	93.6	123.5
W6H6	3.2	64	2.738	0.992	5.46	0.9974	1.13	0.8	5.1
W6H6	3.2	70	2.345	0.998	≥9 (bound)	0.9946	1.70	−2.1	−1.6
W6H6	3.2	76	1.999	1.000	≥9 (bound)	0.9988	0.78	−2.1	−2.1
W6H6	3.2	82	1.664	1.000	≥9 (bound)	0.9985	0.93	−2.1	−2.1

**Table 6:** Measured 1 s creep compliance, linear prediction from the oscillatory data at the same temperature and viscosity ratios. J in kPa^−1^; J_lin_ from the springpot calibration (Equation (9)); Λ from Equation (11) with the springpot (upper bound) and Maxwell (lower bound) calibrations; |η*|/η_creep_ from Equation (12) at ω = 10 rad/s (model-free) and at a frequency matched to the creep shear rate (springpot extrapolation).

Binder	σ_0_ (kPa)	T (°C)	Peak Creep Strain (%)	J_meas_(1 s)	J_lin_(1 s), Springpot	Λ Springpot	Λ Maxwell	|η*|/η_creep_, 10 rad/s	|η*|/η_creep_, Rate-Matched
CONTROL	3.2	64	2210	6.91	4.38	1.58	1.25	1.30	1.17
W4H8	0.1	70	13.4	1.34	0.136	9.90	4.20	1.63	52.36
W4H8	0.1	76	42	4.2	0.103	40.81	21.05	5.12	92.96
W4H8	3.2	64	322	1.01	0.19	5.29	2.20	1.39	2.84
W4H8	3.2	70	921	2.88	0.136	21.23	9.00	3.81	4.01
W4H8	3.2	76	2140	6.69	0.103	64.91	33.48	8.82	4.36
W4H8	3.2	82	4669	14.6	0.176	82.91	44.41	10.92	2.64
W6H6	0.1	64	11.8	1.18	0.208	5.67	2.41	1.51	21.83
W6H6	0.1	70	27.7	2.77	0.197	14.01	5.75	2.73	39.10
W6H6	0.1	76	64.4	6.44	0.187	34.40	15.07	5.40	53.07
W6H6	0.1	82	133	13.3	0.175	76.05	38.61	9.95	61.88
W6H6	3.2	64	591	1.85	0.208	8.86	3.77	2.58	3.51
W6H6	3.2	70	1443	4.51	0.197	22.84	9.38	4.84	3.63
W6H6	3.2	76	3191	9.97	0.187	53.25	23.33	8.86	3.36
W6H6	3.2	82	6840	21.4	0.175	122.09	61.98	16.91	2.96

**Table 7:** Apparent Arrhenius activation energies E_a_ (kJ/mol, ± standard error; coefficient of determination in parentheses) of the oscillatory modulus |G*|, of 1/J_nr,3.2_ and of the springpot constant c of the 3.2 kPa fits. Last column: ratio of the creep (J_nr,3.2_) to the oscillatory (|G*|, 64–82 °C) activation energy.

Binder	|G*|, 52–64 °C	|G*|, 64–82 °C	J_nr,3.2_, 64–82 °C	c (FM3, 3.2 kPa)	E_a_(creep)/E_a_(|G*|)
CONTROL	129 ± <1 (1.00)	— ^a^	136 ± 4 (1.00)	136 ± 4	—
W4H8	85 ± 4 (1.00)	32 ± 15 (0.68)	151 ± 7 (1.00)	149 ± 6	4.78
W6H6	84 ± 6 (0.99)	33 ± 4 (0.97)	138 ± 3 (1.00)	136 ± 2	4.23

^a^ Oscillatory data at 70–82 °C not available (Section 3.1).

**Table 8:** Bootstrap 95% confidence intervals (2000 resamples of the ten cycles; 300 refits for α). α is given with the half-width of its interval; ‘bound’: α = 1 in every resample. Intervals reflect cycle-to-cycle variability within one specimen only.

Binder	σ_0_ (kPa)	T (°C)	J_nr_ (kPa^−1^)	%R	α (FM3)	Λ Springpot
CONTROL	3.2	64	6.96 (6.86–7.05)	−0.7 (−1.0 to −0.5)	1.000 (bound)	1.58 (1.55–1.60)
CONTROL	3.2	70	17.62 (17.46–17.79)	−1.5 (−1.8 to −1.2)	1.000 (bound)	—
CONTROL	3.2	76	40.04 (39.70–40.45)	−2.5 (−2.8 to −2.2)	1.000 (bound)	—
CONTROL	3.2	82	81.85 (80.02–83.67)	−4.4 (−4.7 to −4.1)	1.000 (bound)	—
W4H8	0.1	70	1.28 (1.23–1.33)	4.4 (3.9 to 4.8)	0.995 ± 0.001	9.90 (9.44–10.34)
W4H8	0.1	76	3.43 (3.40–3.47)	18.4 (18.1 to 18.7)	0.941 ± 0.001	40.81 (40.54–41.10)
W4H8	3.2	64	0.947 (0.917–0.981)	5.8 (4.2 to 7.2)	0.988 ± 0.004	5.29 (5.05–5.52)
W4H8	3.2	70	2.85 (2.70–2.98)	1.0 (0.4 to 1.6)	0.999 ± 0.001	21.23 (20.14–22.23)
W4H8	3.2	76	6.72 (6.63–6.81)	−0.5 (−0.8 to −0.1)	1.000 (bound)	64.91 (63.93–65.85)
W4H8	3.2	82	14.76 (14.60–14.92)	−1.2 (−1.4 to −0.9)	1.000 (bound)	82.91 (81.96–83.89)
W6H6	0.1	64	0.778 (0.756–0.802)	34.1 (33.2 to 35.0)	0.879 ± 0.003	5.67 (5.58–5.76)
W6H6	0.1	70	2.16 (2.13–2.18)	22.0 (21.7 to 22.4)	0.929 ± 0.001	14.01 (13.90–14.12)
W6H6	0.1	76	5.61 (5.56–5.65)	12.9 (12.7 to 13.2)	0.960 ± 0.001	34.40 (34.17–34.61)
W6H6	0.1	82	12.56 (12.47–12.68)	5.6 (4.6 to 6.3)	0.985 ± 0.002	76.05 (75.75–76.36)
W6H6	3.2	64	1.79 (1.73–1.84)	3.0 (2.1 to 3.7)	0.992 ± 0.003	8.86 (8.56–9.10)
W6H6	3.2	70	4.50 (4.36–4.65)	0.3 (0.0 to 0.7)	0.998 ± 0.001	22.84 (22.11–23.64)
W6H6	3.2	76	10.03 (9.93–10.12)	−0.6 (−0.9 to −0.4)	1.000 (bound)	53.25 (52.75–53.70)
W6H6	3.2	82	21.72 (21.62–21.81)	−1.6 (−1.9 to −1.3)	1.000 (bound)	122.09 (121.62–122.74)

## Data Availability

The raw DSR and MSCR data files and the analysis scripts supporting the findings of this study are available from the corresponding author upon reasonable request.

## Supplementary Materials

The following supporting information can be downloaded at https://www.mdpi.com/article/10.3390/polym18212785/s1. References [10,12,18,19,22,23,29,30,31,32,33,35,37,40,41,42,43,72,73,74,75] are cited in the Supplementary Materials.

## Abbreviations

The following abbreviations are used in this manuscript:

Abbreviation	Meaning
AASHTO	American Association of State Highway and Transportation Officials
AICc	Corrected Akaike information criterion
ASTM	American Society for Testing and Materials
CI	Confidence interval
CONTROL	Unmodified (base) binder
DSR	Dynamic shear rheometer
FM3	Three-parameter fractional Maxwell model
FM4	Four-parameter fractional model
HDPE	High-density polyethylene
MSCR	Multiple stress creep recovery
NBRRI	Nigerian Building and Road Research Institute
NRMSE	Normalized root-mean-square error
PAV	Pressure aging vessel
PE	Polyethylene
RMS	Root-mean-square
RTFOT	Rolling thin-film oven test
SARA	Saturates, aromatics, resins and asphaltenes
SSE	Sum of squared errors
W4H8	Binder with 4 wt% WEO and 8 wt% HDPE
W6H6	Binder with 6 wt% WEO and 6 wt% HDPE
WEO	Waste engine oil
