# Supplementary material for: Oscillatory Stiffness Versus Creep–Recovery Response of Recycled High-Density Polyethylene/Waste Engine Oil Modified Bitumen
Source: Polymers (Basel). 2026 Nov 7;18(21):2785. doi: 10.3390/polym18212785 (PMC13765835; doi:10.3390/polym18212785)
Supplement: Supplementary file 1 [file polymers-18-02785-s001.zip › polymers-4674852-supplementary.pdf]

polymers

MDPI

*Supplementary Materials*

# Oscillatory Stiffness Versus Creep–Recovery Response of Recycled High-Density Polyethylene/Waste Engine Oil Modified Bitumen

**Hassan Shuaibu Abdulrahman, Michael Chiahemba Iorpenda, Muhammed Fatih Yentimur, Ali Almusawi and Mustafa Albdairi**

## S1. Literature Summary

Tables S1–S3 and Figures S1 and S2 summarize the binder-level studies referred to in the Introduction of the main text. Studies are cited by author and year; full references are listed at the end of this document.

**Table S1.** Binder-level studies of polyolefin-modified bitumen: oscillatory and creep findings.

| Study | Base binder | Polyolefin (wt%) | Ageing before creep test | Oscillatory finding | Creep, MSCR or steady-shear finding |
|---|---|---|---|---|---|
| Pérez-Lepe et al. 2003 | 60/70, 150/200 | HDPE, LDPE (5) | No creep test | HDPE: G′ > G″ and low-frequency plateau at 75 °C (rotor–stator mixing) | HDPE shear-thinning without Newtonian plateau; Cox–Merz rule not obeyed (obeyed for LDPE, SBS) |
| Cardone et al. 2014 | 70/100 | Polyolefin (2–6), peroxide | No creep test | 6 wt%: \|G*\| plateau; δ falls above 40 °C | Cox–Merz rule holds at 2 wt% only |
| González et al. 2016 | 70/100 | Recycled HDPE (5) | Unaged | SAOS does not separate the PMBs | LAOS at 20 °C: HDPE most strain-sensitive; nonlinear from 1.4–4.1% strain |
| Saroufim et al. 2018 | 50/70 | HDPE (2.5, 5), LDPE, EVA (5) | No creep test | 5% HDPE: low-frequency plateau (filler type) | Flow curves: low-shear upturn for 5% HDPE only |
| Nizamuddin et al. 2020 | C320 (≈50/70) | Recycled LLDPE (3–12) | Not stated | Black-diagram plateau, δ ≈ 82° → 54° with dosage | %R up and $J_{nr}$ down with dosage (graphs only) |
| Wang et al. 2022 | 70/100 | Waste PE pellets, shreds (5) | Unaged | δ falls at high T in most laboratories; poor reproducibility | No creep test |
| Tušar et al. 2023 | 70/100 | Waste PE pellets, shreds (5) | Unaged | PG 64 → 76 °C | 60 °C: $J_{nr,3.2}$ reduced 4–5×; $\%R_{3.2}$ of one blend 96% and 6% in two laboratories |
| Binti Joohari and Giustozzi 2022 | C170 (≈70/100) | LDPE, LLDPE, recycled LLDPE (2) + 3.5% SBS | Not stated | Not reported | 60 °C: LDPE or LLDPE raised $J_{nr,3.2}$ of the SBS binder from 0.33 to 0.82–0.83 kPa$^{-1}$ and cut $\%R_{3.2}$ by about 60% |
| Singh et al. 2023 | VG-30 | HDPE, post-consumer or post-industrial (1.5) | USAT + PAV | PG 64 → 82 and 94 °C | 60 °C: $J_{nr,3.2}$ reduced 9× and about 100× |

 https://doi.org/10.3390/polym18212785

| Study | Base binder | Polyolefin (wt%) | Ageing before creep test | Oscillatory finding | Creep, MSCR or steady-shear finding |
|---|---|---|---|---|---|
| Singh and Gupta 2025 | VG-30 | LDPE + S or MAH, 75:25 (1.5–7.5) | USAT | PG 64 → 70–88 °C | 60 °C, 0.1–12.8 kPa: $J_{nr}$ rises over the first ~30 cycles at 3.2 kPa; ranking agrees with mixtures only at ≥ 6.4 kPa |

Abbreviations: PE, polyethylene; HDPE, LDPE and LLDPE, high-density, low-density and linear low-density PE; EVA, ethylene–vinyl acetate; SBS, styrene–butadiene–styrene; MAH, maleic anhydride; S, sulfur; PMB, polymer-modified bitumen; PG, performance grade; USAT, universal simple aging test; PAV, pressure aging vessel; SAOS and LAOS, oscillatory shear in the linear and in the nonlinear amplitude range; C170, C320, VG-30 and 60/70, 70/100, 150/200: viscosity and penetration grades of the base binders.

**Table S2.** Binder-level studies combining a polyolefin with an oil, and the present study.

| Study | Binder; plastic (wt%); oil (wt%) | Ratio design | Ageing | Tests | Key finding |
|---|---|---|---|---|---|
| Luo et al. 2017 | 80/100; LLDPE 3–5 + SBS 3.5–5.5; waste cooking oil 5 | Not varied | TFOT | T and f sweeps; repeated creep, 100 Pa (Burgers) | Oil improves compatibility (ΔSP 9.5 → 2.4 °C), slightly lowers ∣G*∣ |
| Fernandes et al. 2018 | 35/50; recycled HDPE 6; engine oil or bottoms 10, 15 (bitumen replacement) | HDPE fixed, oil varied; total not constant | RTFOT (mass loss only) | Master curves; MSCR 0.025–3.2 kPa, 64 °C | PG 76 °C; HDPE + engine oil $J_{nr}$ above commercial PMB above 0.4 kPa |
| Nouali et al. 2021 | 40/50; HDPE bag waste 0–2; used engine oil 2 | Oil fixed | RTFOT (conventional) | Conventional tests, nomograph | Oil raises dissolvable plastic to 2 wt%; complete separation in storage |
| El-Sherbeni et al. 2025 | Binder recovered from RAP; recycled LDPE 2; WEO 15 (rejuvenator) | One combination | RTFO | DSR, MSCR, LAS | PG 64 → 82 °C; $J_{nr,3.2}$ 2.79 → 1.95 $kPa^{-1}$ |
| Li et al. 2026 | AH-70; waste HDPE, LDPE, LLDPE 2 and LDPE 2–8; WEO 6 | Oil fixed, PE varied (total 8–14) | Unaged | T sweeps (12% strain), f sweeps, BBR, healing | δ rises then falls with T; optimum 2% LDPE + 6% WEO; no MSCR |
| This study | 60/70; recycled HDPE 8 and 6; WEO 4 and 6 | HDPE:WEO 2:1 and 1:1 at 12 wt% total | PAV | Oscillatory DSR 52–82 °C; MSCR 64–82 °C; fractional modelling | See main text, Sections 3 and 4 |

Abbreviations: TFOT, thin-film oven test; RTFOT or RTFO, rolling thin-film oven test; PAV, pressure aging vessel; RAP, reclaimed asphalt pavement; WEO, waste engine oil; LAS, linear amplitude sweep; BBR, bending beam rheometer; ΔSP, softening-point difference after storage; T and f, temperature and frequency; other abbreviations as in Table S1.

**Table S3.** Constitutive models fitted to creep–recovery or oscillatory data of asphalt binders.

| Study | Model (parameters) | Binders | Data fitted | Use of oscillatory data | Main finding |
|---|---|---|---|---|---|
| Luo et al. 2017 | Burgers (4) | SBS/LLDPE + waste cooking oil | Repeated creep, 100 Pa, 60–70 °C, cycles 50–51 | None | Viscous component $G_v$ ranks the binders |

| Study | Model (parameters) | Binders | Data fitted | Use of oscillatory data | Main finding |
|---|---|---|---|---|---|
| Laukkanen and Winter 2018 | Linear viscoelastic spectrum with Boltzmann superposition | Unmodified and modified | Repeated creep–recovery | Predicts the creep from SAOS | Agreement within about 20% for modified binders |
| Baglieri et al. 2017 | Springpot + dashpot (3) | SBS 3 and 6% (RTFOT) | Single creep–recovery, 100 Pa, 900 + 900 s, 40–80 °C | None | $\alpha$ 0.55–0.75 at 40 °C and 1 at 60 °C (3% SBS); 0.10–0.26 at 60 °C (6% SBS) |
| Hajikarimi et al. 2018 | Sum of power laws with stress exponent (5; up to 10 with recovery) | SBS, CR, PPA | Creep–recovery, 0.1–30 kPa, 40 °C | None | Nonlinearity index m; CR and PPA amplify nonlinearity |
| Jafari et al. 2025 | As, genetic-algorithm fit (5) | SBS, PPA (RTFOT) | MSCR cycles, 0.1–40 kPa, 40–70 °C | Only to match PG | Binders with equal PG differ above 12.8 kPa |
| Mishra et al. 2026 | Fractional Maxwell (3) | Crumb rubber 5–30% (unaged, RTFOT, PAV) | Frequency sweeps, 0.1–100 rad/s, 10–70 °C (G′, G″) | Fitted to oscillation only; no creep | $R^2 > 0.96$ in 50 of 53 data sets; $\alpha$ rises almost linearly with T, less at high CR content |
| This study | Fractional Maxwell (3), two-springpot (4), Burgers (4); AICc | HDPE/WEO (PAV) | Mean MSCR cycles, 0.1 and 3.2 kPa, 64–82 °C | Springpot and Maxwell calibrations predict the 1-s creep ($\Lambda$) | See main text, Sections 3.3–3.5 |

Abbreviations: CR, crumb rubber; PPA, polyphosphoric acid; $G_v$, viscous component of the Burgers model; AICc, corrected Akaike information criterion; other abbreviations as in Tables S1 and S2.

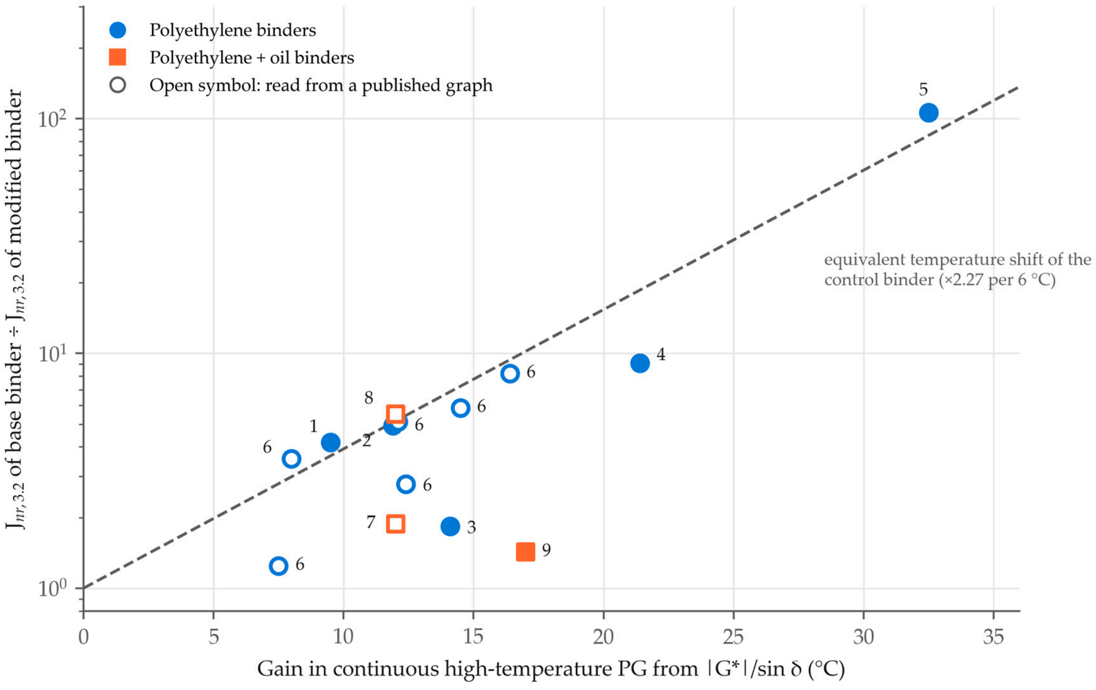


**Figure S1.** Gain in continuous high-temperature grade, from |G*|/sin δ, against the reduction of $J_{nr,3.2}$ relative to the base binder in published studies. 1, 2: Tušar et al. 2023, 5 wt% waste PE pellets and shreds, unaged, 60 °C, laboratory 10; 3: El-Sherbeni et al. 2025, 2 wt% recycled LDPE, RTFO, temperature not reported; 4, 5: Singh et al. 2023, 1.5 wt% post-consumer and post-industrial HDPE,

https://doi.org/10.3390/polym18212785

USAT + PAV, 60 °C; 6: Singh and Gupta 2025, 1.5–4.5 wt% LDPE with sulfur or maleic anhydride, USAT, 60 °C; 7, 8: Fernandes et al. 2018, 6 wt% HDPE with 10 wt% engine oil or 15 wt% recycled engine-oil bottoms, unaged, 64 °C, with a grade step of 12 °C used as the gain; 9: El-Sherbeni et al. 2025, RAP binder rejuvenated with 15 wt% WEO plus 2 wt% LDPE. Open symbols were read from published graphs and are approximate. Dashed line: $J_{nr,3.2}$ reduction expected from a pure temperature shift of the control binder of the present study, a factor of 2.27 per 6 °C. The data come from different base binders, aging protocols and test temperatures and can be compared only to within an order of magnitude.

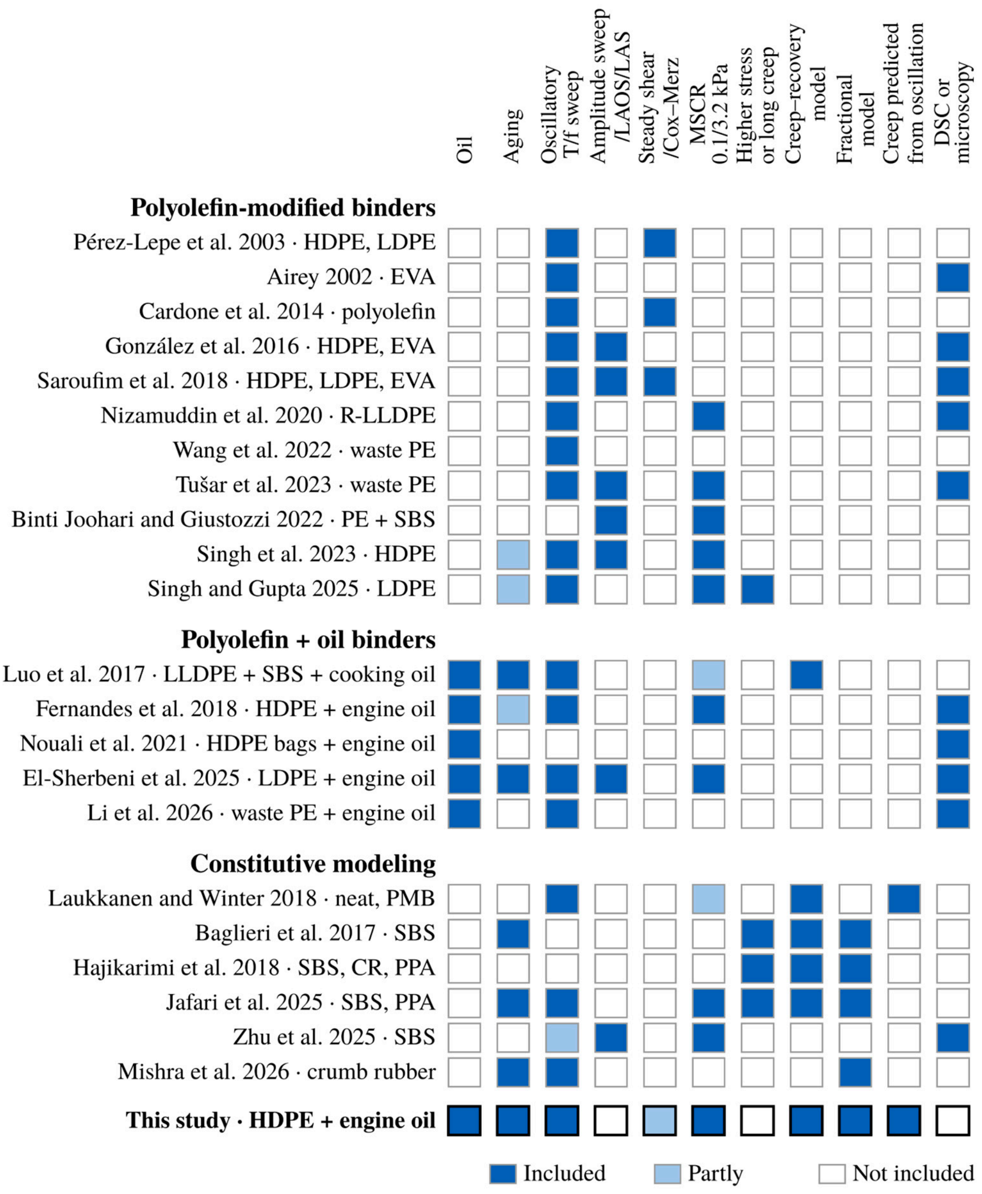


**Figure S2.** Test methods used in the studies reviewed and in the present study. Constitutive modeling includes models fitted to oscillatory data (Mishra et al. 2026). Partly: short-term aging by USAT or RTFOT used for mass loss only (Fernandes et al. 2018; Singh et al. 2023; Singh and Gupta 2025); repeated creep at a single stress (Luo et al. 2017; Laukkanen and Winter 2018); single-point oscillation (Zhu et al. 2025); creep-derived rather than steady-shear viscosity in the present study. For the present study the aging column refers to PAV aging.

https://doi.org/10.3390/polym18212785

## S2. Data of the PAV-Aged Binders

Tables S4–S6 give the numerical values of the oscillatory, MSCR and structural-breakdown data shown in the figures of Section 3 of the main text, and Figure S3 gives the complete strain–time records of all MSCR tests.

**Table S4.** Oscillatory data of the PAV-aged binders at 10 rad/s: $|G^*|$ in kPa and δ in degrees.

| T (°C) | CONTROL | | W4H8 | | W6H6 | |
|---|---|---|---|---|---|---|
| | $\|G^*\|$ | δ | $\|G^*\|$ | δ | $\|G^*\|$ | δ |
| 52 | 9.88 | 72.7 | 42.3 | 52.0 | 42.0 | 51.6 |
| 58 | 4.15 | 76.7 | 22.9 | 43.0 | 22.2 | 47.9 |
| 64 | 1.81 | 79.1 | 13.9 | 33.4 | 13.9 | 36.9 |
| 70 | — [a] | — [a] | 13.0 | 18.7 | 10.4 | 24.2 |
| 76 | — [a] | — [a] | 12.9 | 9.1 | 8.70 | 16.0 |
| 82 | — [a] | — [a] | 7.33 | 8.2 | 7.70 | 9.6 |

[a] Not reported: measured at torques of 0.07–0.25 μN·m, close to the lower limit of the instrument.

**Table S5.** MSCR results of the PAV-aged binders, mean of ten cycles. $J_{nr}$ in $kPa^{-1}$; %R and $J_{nr,diff}$ in %.

| Binder | T (°C) | $J_{nr,0.1}$ | $J_{nr,3.2}$ | $J_{nr,diff}$ | $\%R_{0.1}$ | $\%R_{3.2}$ |
|---|---|---|---|---|---|---|
| CONTROL | 64 | — [b] | 6.96 | — | — | -0.7 |
| CONTROL | 70 | — [b] | 17.62 | — | — | -1.5 |
| CONTROL | 76 | — [b] | 40.04 | — | — | -2.5 |
| CONTROL | 82 | — [b] | 81.85 | — | — | -4.4 |
| W4H8 | 64 | — [c] | 0.95 | — | — | 5.8 |
| W4H8 | 70 | 1.28 | 2.85 | 122 | 4.4 | 1.0 |
| W4H8 | 76 | 3.43 | 6.72 | 96 | 18.4 | -0.5 |
| W4H8 | 82 | — [d] | 14.76 | — | — | -1.2 |
| W6H6 | 64 | 0.78 | 1.79 | 130 | 34.1 | 3.0 |
| W6H6 | 70 | 2.16 | 4.50 | 108 | 22.0 | 0.3 |
| W6H6 | 76 | 5.61 | 10.03 | 79 | 12.9 | -0.6 |
| W6H6 | 82 | 12.56 | 21.72 | 73 | 5.6 | -1.6 |

[b] Not reported: the 0.1 kPa stage of the control was inconsistent with its 3.2 kPa stage, with compliances 18–540 times lower and a negative Jnr at 76 °C; treated as a measurement effect. [c] Not reported: recovery exceeded the creep strain (%R > 100%, Jnr < 0), which is physically impossible; treated as a measurement effect. [d] Not reported: between-cycle coefficient of variation of the peak strain 31%.

**Table S6.** Indicators of structural breakdown from the MSCR data: apparent creep viscosity $\eta_{creep}$ and creep shear rate $\dot{\gamma}$ at the two stress levels, flow index n (main text, Section 2.8) and recoverable strain $\varepsilon_c - \varepsilon_r$ of each stage. —: stage not available (Table S5).

| Binder | T (°C) | $\eta_{creep,0.1}$ (Pa·s) | $\eta_{creep,3.2}$ (Pa·s) | $\dot{\gamma}_{0.1}$ ($s^{-1}$) | $\dot{\gamma}_{3.2}$ ($s^{-1}$) | n | $\varepsilon_c - \varepsilon_r$, 0.1 kPa (%) | $\varepsilon_c - \varepsilon_r$, 3.2 kPa (%) |
|---|---|---|---|---|---|---|---|---|
| CONTROL | 64 | — | 139 | — | 23.0 | — | — | <0 [f] |
| CONTROL | 70 | — | 56 | — | 57.2 | — | — | <0 [f] |
| CONTROL | 76 | — | 24 | — | 131.0 | — | — | <0 [f] |
| CONTROL | 82 | — | 12 | — | 271.6 | — | — | <0 [f] |
| W4H8 | 64 | — | 1,000 | — | 3.2 | — | — | 18.8 |
| W4H8 | 70 | 795 | 341 | 0.13 | 9.4 | 0.80 | 0.6 | 9.6 |

https://doi.org/10.3390/polym18212785

| Binder | T (°C) | $\eta_{creep,0.1}$ (Pa·s) | $\eta_{creep,3.2}$ (Pa·s) | $\dot{\gamma}_{0.1}$ ($s^{-1}$) | $\dot{\gamma}_{3.2}$ ($s^{-1}$) | n | $\varepsilon_c - \varepsilon_r$, 0.1 kPa (%) | $\varepsilon_c - \varepsilon_r$, 3.2 kPa (%) |
|---|---|---|---|---|---|---|---|---|
| W4H8 | 76 | 252 | 146 | 0.40 | 21.9 | 0.86 | 7.7 | <0[f] |
| W4H8 | 82 | — | 67 | — | 47.7 | — | — | <0[f] |
| W6H6 | 64 | 918 | 539 | 0.11 | 5.9 | 0.87 | 4.0 | 17.5 |
| W6H6 | 70 | 382 | 216 | 0.26 | 14.8 | 0.86 | 6.1 | 4.6 |
| W6H6 | 76 | 161 | 98 | 0.62 | 32.6 | 0.87 | 8.3 | <0[f] |
| W6H6 | 82 | 77 | 46 | 1.29 | 70.3 | 0.87 | 7.5 | <0[f] |

[f] Negative: strain continued to increase after unloading (main text, Section 3.2).

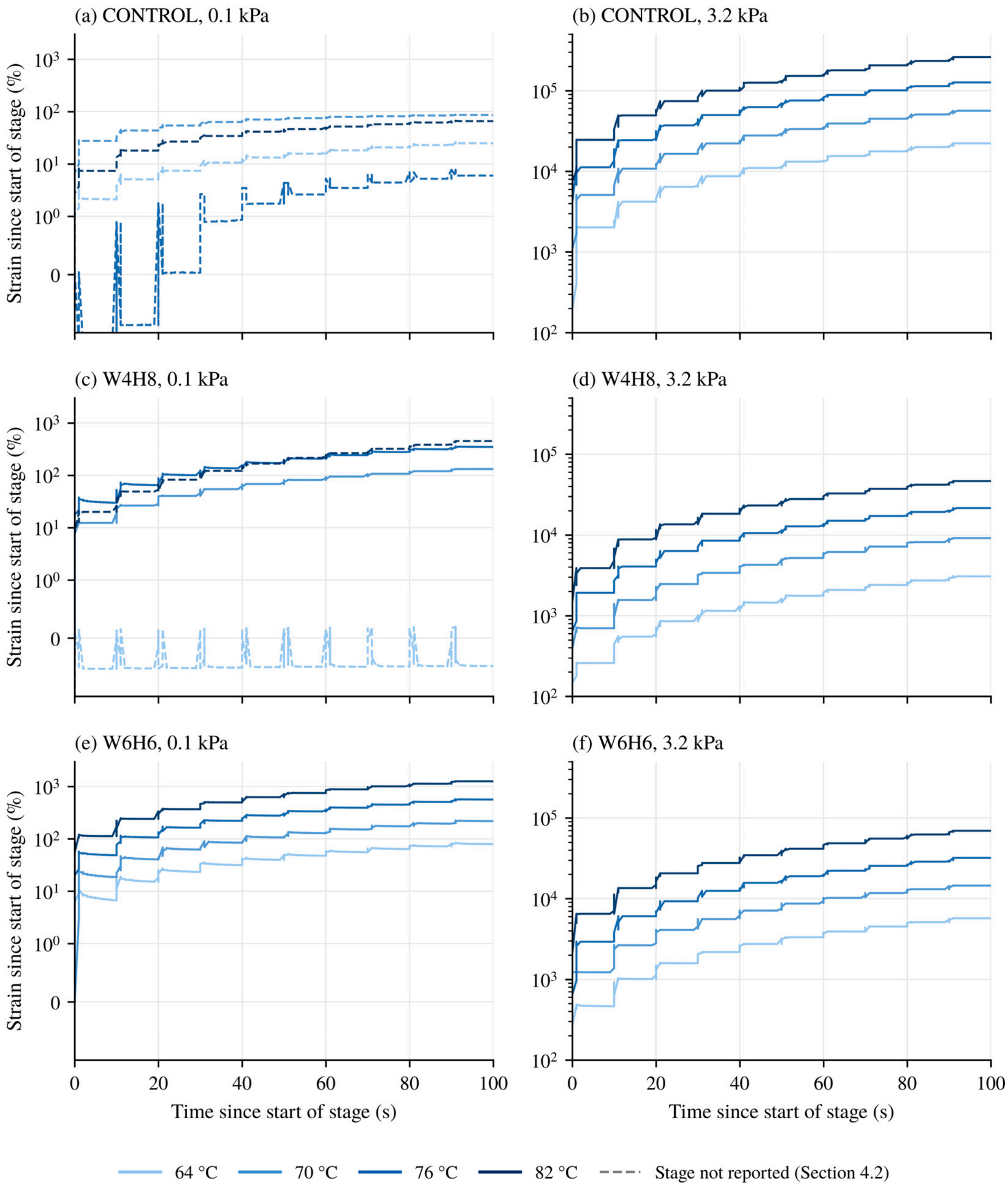


**Figure S3.** Complete strain–time records of all MSCR tests of the PAV-aged binders, as strain since the start of each stage of ten creep–recovery cycles, each of 1 s creep and 9 s recovery: (a,c,e) 0.1 kPa; (b,d,f) 3.2 kPa. Dashed lines mark the stages that were not reported in the main text (Section 3.2). The vertical axis of the 0.1 kPa panels is logarithmic above 1% and linear below.

## S3. Validation and Parameter Sensitivity of the Fractional Maxwell Model

Tables S7 and S8 give the validation and sensitivity results of the three-parameter fractional Maxwell model summarized in Section 3.3 of the main text.

**Table S7.** Validation of the FM3 and Burgers models for each condition: error in $J_{nr}$, (predicted − measured)/measured in %, and NRMSE of the recovery phase predicted from the creep phase. Cycle split: fitted to cycles 1–5, predicted cycles 6–10. Phase split: fitted to 0–1 s, predicted 1–10 s. Temperature split: Arrhenius relation of c fitted to the other three temperatures (3.2 kPa only).

| Binder | $\sigma_0$ (kPa) | T (°C) | Cycle split, FM3 | Cycle split, naive[a] | Phase split, FM3 | Phase split, Burgers | Recovery NRMSE, FM3 (%) | Recovery NRMSE, Burgers (%) | Temperature split, FM3 |
|---|---|---|---|---|---|---|---|---|---|
| CONTROL | 3.2 | 64 | −1.4 | −1.3 | −2.3 | −2.3 | 2.49 | 2.49 | 14.2 |
| CONTROL | 3.2 | 70 | −1.6 | −1.5 | −2.9 | −2.9 | 3.16 | 3.16 | −5.6 |
| CONTROL | 3.2 | 76 | 0.7 | 0.8 | −4.5 | −4.5 | 4.79 | 4.79 | −5.6 |
| CONTROL | 3.2 | 82 | −6.3 | −6.0 | −7.5 | −7.5 | 7.96 | 7.96 | 13.5 |
| W4H8 | 0.1 | 70 | 11.0 | 11.2 | −17.0 | −24.4 | 13.45 | 23.26 | — |
| W4H8 | 0.1 | 76 | −0.1 | 0.5 | −1.3 | 4.6 | 0.76 | 2.42 | — |
| W4H8 | 3.2 | 64 | −9.2 | −9.1 | 2.1 | 5.3 | 2.45 | 4.78 | 27.9 |
| W4H8 | 3.2 | 70 | −11.4 | −11.5 | 0.2 | 0.2 | 0.17 | 0.17 | −11.3 |
| W4H8 | 3.2 | 76 | −1.3 | −1.2 | −1.4 | −1.4 | 1.58 | 1.58 | −5.9 |
| W4H8 | 3.2 | 82 | 2.7 | 2.7 | −2.1 | −2.1 | 2.25 | 2.25 | 15.7 |
| W6H6 | 0.1 | 64 | 7.7 | 6.9 | 11.0 | 29.0 | 6.42 | 14.71 | — |
| W6H6 | 0.1 | 70 | −0.3 | −0.5 | 3.1 | 12.9 | 2.34 | 7.25 | — |
| W6H6 | 0.1 | 76 | −0.9 | −0.8 | −0.5 | 0.1 | 0.54 | 2.02 | — |
| W6H6 | 0.1 | 82 | −1.4 | −0.7 | −5.6 | −4.1 | 3.93 | 4.23 | — |
| W6H6 | 3.2 | 64 | −6.1 | −6.0 | 2.8 | 2.8 | 2.64 | 2.64 | 9.7 |
| W6H6 | 3.2 | 70 | −2.6 | −2.7 | −0.9 | −0.9 | 1.06 | 1.06 | −4.6 |
| W6H6 | 3.2 | 76 | −1.2 | −1.1 | −1.5 | −1.5 | 1.70 | 1.70 | −1.8 |
| W6H6 | 3.2 | 82 | 0.6 | 0.7 | −2.7 | −2.7 | 2.92 | 2.92 | 4.5 |

[a] Naive prediction: mean of cycles 1–5 used directly as the prediction for cycles 6–10, i.e. the within-test change between the two halves of the stage.

**Table S8.** Parameter sensitivity of the FM3 fits to the mean MSCR cycles. SE: standard error from the Jacobian of the fit; r: correlation coefficient between parameters; '≥9 (bound)': spring not identifiable. The elasticity of $J_{nr}$ with respect to c was −0.99 in every case. $\Delta J_{nr}$ and Δ%R: change of $J_{nr}$ (in %) and of %R (in percentage points, pp) for a 1% change of $\alpha$.

| Binder | $\sigma_0$ (kPa) | T (°C) | log c ± SE | $\alpha$ ± SE | log G ± SE | r(c, $\alpha$) | r(G, $\alpha$) | $\Delta J_{nr}$ per 1% $\alpha$ (%) | Δ%R per 1% $\alpha$ (pp) |
|---|---|---|---|---|---|---|---|---|---|
| CONTROL | 3.2 | 64 | 2.158 ± 0.001 | 1.000 ± 0.001 | ≥9 (bound) | 0.91 | — | 2.80 | −3.20 |
| CONTROL | 3.2 | 70 | 1.754 ± 0.001 | 1.000 ± 0.001 | ≥9 (bound) | 0.91 | — | 2.80 | −3.20 |
| CONTROL | 3.2 | 76 | 1.398 ± 0.001 | 1.000 ± 0.001 | ≥9 (bound) | 0.91 | — | 2.80 | −3.20 |
| CONTROL | 3.2 | 82 | 1.088 ± 0.002 | 1.000 ± 0.002 | ≥9 (bound) | 0.91 | — | 2.80 | −3.20 |
| W4H8 | 0.1 | 70 | 2.892 ± 0.001 | 0.999 ± 0.001 | 4.23 ± 0.02 | 0.94 | −0.58 | 2.80 | −3.04 |
| W4H8 | 0.1 | 76 | 2.395 ± 0.001 | 0.942 ± 0.001 | 4.25 ± 0.05 | 0.94 | −0.62 | 2.79 | −2.57 |
| W4H8 | 3.2 | 64 | 3.009 ± <0.001 | 0.988 ± <0.001 | 4.92 ± 0.04 | 0.94 | −0.58 | 2.84 | −3.10 |
| W4H8 | 3.2 | 70 | 2.544 ± <0.001 | 0.999 ± <0.001 | ≥9 (bound) | 0.91 | — | 2.80 | −3.19 |
| W4H8 | 3.2 | 76 | 2.173 ± <0.001 | 1.000 ± <0.001 | ≥9 (bound) | 0.91 | — | 2.80 | −3.20 |
| W4H8 | 3.2 | 82 | 1.831 ± 0.001 | 1.000 ± 0.001 | ≥9 (bound) | 0.91 | — | 2.80 | −3.20 |
| W6H6 | 0.1 | 64 | 2.950 ± <0.001 | 0.879 ± <0.001 | ≥9 (bound) | 0.87 | — | 2.71 | −2.02 |
| W6H6 | 0.1 | 70 | 2.575 ± <0.001 | 0.928 ± <0.001 | 4.89 ± 0.08 | 0.93 | −0.63 | 2.77 | −2.45 |
| W6H6 | 0.1 | 76 | 2.204 ± <0.001 | 0.961 ± <0.001 | 4.21 ± 0.04 | 0.94 | −0.60 | 2.81 | −2.80 |

https://doi.org/10.3390/polym18212785

| Binder | $\sigma_0$ (kPa) | T (°C) | log c ± SE | α ± SE | log G ± SE | r(c, α) | r(G, α) | $\Delta J_{nr}$ per 1% α (%) | Δ%R per 1% α (pp) |
|---|---|---|---|---|---|---|---|---|---|
| W6H6 | 0.1 | 82 | 1.885 ± 0.001 | 0.985 ± 0.001 | 3.71 ± 0.05 | 0.94 | −0.59 | 2.84 | −3.06 |
| W6H6 | 3.2 | 64 | 2.740 ± <0.001 | 0.994 ± <0.001 | 5.23 ± 0.08 | 0.94 | −0.58 | 2.79 | −3.12 |
| W6H6 | 3.2 | 70 | 2.347 ± <0.001 | 1.000 ± <0.001 | ≥9 (bound) | 0.91 | — | 2.80 | −3.20 |
| W6H6 | 3.2 | 76 | 1.999 ± <0.001 | 1.000 ± <0.001 | ≥9 (bound) | 0.91 | — | 2.80 | −3.20 |
| W6H6 | 3.2 | 82 | 1.664 ± 0.001 | 1.000 ± 0.001 | ≥9 (bound) | 0.91 | — | 2.80 | −3.20 |

## S4. Base Bitumen Before Aging

Table S9 gives the data of the base bitumen tested before aging with the same oscillatory and MSCR protocols as the aged binders. These data are used in the main text only as an additional linear reference for the creep prediction (Sections 3.2 and 3.4).

**Table S9.** Base bitumen before aging: |G*| and δ at 10 rad/s, MSCR results as the mean of ten cycles, and ratio Λ of the measured to the linearly predicted 1-s creep compliance with the springpot and Maxwell calibrations, given as springpot/Maxwell. $J_{nr}$ in $kPa^{-1}$; $J_{nr,diff}$ and $\%R_{3.2}$ in %. $\%R_{0.1}$ ranged from −1.6% at 64 °C to −10.0% at 82 °C.

| T (°C) | \|G*\| (kPa) | δ (°) | $J_{nr,0.1}$ | $J_{nr,3.2}$ | $J_{nr,diff}$ | $\%R_{3.2}$ | Λ, 0.1 kPa | Λ, 3.2 kPa |
|---|---|---|---|---|---|---|---|---|
| 64 | 0.261 | 87.2 | 39.3 | 50.1 | 27.5 | −3.2 | 1.07/1.01 | 1.35/1.26 |
| 70 | 0.139 | 87.5 | 76.0 | 92.2 | 21.3 | −5.0 | 1.07/1.01 | 1.29/1.22 |
| 76 | 0.078 | 87.0 | 133.9 | 159.7 | 19.3 | −8.6 | 1.05/0.98 | 1.22/1.14 |
| 82 | 0.046 | 86.3 | 219.5 | 250.4 | 14.1 | −12.4 | 0.99/0.91 | 1.11/1.02 |

## References


Baglieri, O., Santagata, E., Sapora, A., Cornetti, P., & Carpinteri, A. (2017). Fractional viscoelastic modeling of antirutting response of bituminous binders. *Journal of Engineering Mechanics*, *143*(5), D4016002. https://doi.org/10.1061/(ASCE)EM.1943-7889.0001081.

Joohari, I. B., & Giustozzi, F. (2022). Oscillatory shear rheometry of hybrid polymer-modified bitumen using multiple stress creep and recovery and linear amplitude sweep tests. *Construction and Building Materials*, *315*, 125791. https://doi.org/10.1016/j.conbuildmat.2021.125791.

Cardone, F., Ferrotti, G., Frigio, F., & Canestrari, F. (2014). Influence of polymer modification on asphalt binder dynamic and steady flow viscosities. *Construction and Building Materials*, *71*, 435-443. https://doi.org/10.1016/j.conbuildmat.2014.08.043.

El-Sherbeni, A. A., Gabr, A. R., El-Badawy, S. M., & Awed, A. M. (2025). Multifaceted assessment of modifying and rejuvenating asphalt binders with recycled waste plastic and engine oil. *Scientific Reports*, *15*(1), 43013. https://doi.org/10.1038/s41598-025-25951-z.

Fernandes, S. R., Silva, H. M., & Oliveira, J. R. (2018). Developing enhanced modified bitumens with waste engine oil products combined with polymers. *Construction and Building Materials*, *160*, 714-724. https://doi.org/10.1016/j.conbuildmat.2017.11.112.

González, Emma, Liliana MB Costa, Hugo MRD Silva, and Loic Hilliou. "Rheological characterization of EVA and HDPE polymer modified bitumens under large deformation at 20 °C." *Construction and Building Materials* 112 (2016): 756-764. https://doi.org/10.1016/j.conbuildmat.2016.02.192

Hajikarimi, P., Nejad, F. M., Khodaii, A., & Fini, E. H. (2018). Introducing a stress-dependent fractional nonlinear viscoelastic model for modified asphalt binders. *Construction and Building Materials*, *183*, 102-113. https://doi.org/10.1016/j.conbuildmat.2018.06.166.

Jafari, M., Ehsani, M., Hajikarimi, P., & Moghadas Nejad, F. (2025). Nonlinear fractional viscoelastic modeling of high-temperature rheological behaviour of SBS and PPA modified asphalt binders. *International Journal of Pavement Engineering*, *26*(1), 2487614. https://doi.org/10.1080/10298436.2025.2487614.

Laukkanen, O. V., & Winter, H. H. (2018). Strain accumulation in bituminous binders under repeated creep-recovery loading predicted from small-amplitude oscillatory shear (saos) experiments. *Mechanics of Time-Dependent Materials*, *22*(4), 499-518. https://doi.org/10.1007/s11043-017-9373-9.

Li, W., Gao, Y., Zhan, M., Zhu, J., & Zhen, S. (2026). Rheological characteristics, self-healing performance, and modification mechanism of bituminous binder modified by waste polyethylene and waste engine oil. *Materials and Structures*, *59*(7), 327. https://doi.org/10.1617/s11527-026-03218-x.

Luo, W., Zhang, Y., & Cong, P. (2017). Investigation on physical and high temperature rheology properties of asphalt binder adding waste oil and polymers. *Construction and Building Materials*, *144*, 13-24. https://doi.org/10.1016/j.conbuildmat.2017.03.105.

Mishra, A., Jain, S., Wang, H., Airey, G., & Sreeram, A. (2026). Revisiting thermorheological simplicity in crumb rubber modified bitumen using fractional Maxwell modelling. *Mechanics of Time-Dependent Materials*, *30*(4), 80. https://doi.org/10.1007/s11043-026-09927-8.

Nizamuddin, S., Jamal, M., Gravina, R., & Giustozzi, F. (2020). Recycled plastic as bitumen modifier: The role of recycled linear low-density polyethylene in the modification of physical, chemical and rheological properties of bitumen. *Journal of Cleaner Production*, *266*, 121988. https://doi.org/10.1016/j.jclepro.2020.121988.

Nouali, M., Derriche, Z., & Ghorbel, E. (2021). Effect of used engine oil addition on the rheological properties of a plastic bag waste–modified 40/50 grade bitumen. *Journal of Materials in Civil Engineering*, *33*(3), 04020484. https://doi.org/10.1061/(ASCE)MT.1943-5533.0003533.

Pérez-Lepe, A., Martınez-Boza, F. J., Gallegos, C., González, O., Muñoz, M. E., & Santamarıa, A. (2003). Influence of the processing conditions on the rheological behaviour of polymer-modified bitumen☆. *Fuel*, *82*(11), 1339-1348. https://doi.org/10.1016/S0016-2361(03)00065-6.

Saroufim, E., Celauro, C., & Mistretta, M. C. (2018). A simple interpretation of the effect of the polymer type on the properties of PMBs for road paving applications. *Construction and Building Materials*, *158*, 114-123. https://doi.org/10.1016/j.conbuildmat.2017.10.034.

Singh, A., Gupta, A., & Miljković, M. (2023). Intermediate-and high-temperature damage of bitumen modified by HDPE from various sources. *Road Materials and Pavement Design*, *24*(sup1), 640-653. https://doi.org/10.1080/14680629.2023.2181017.

Singh, A., & Gupta, A. (2025). Revisiting MSCR test parameters for LDPE-modified asphalt binders. *Construction and Building Materials*, *494*, 143353. https://doi.org/10.1016/j.conbuildmat.2025.143353.

Tušar, M., Kakar, M. R., Poulikakos, L. D., Pasquini, E., Baliello, A., Pasetto, M., ... & Carter, A. (2023). RILEM TC 279 WMR round robin study on waste polyethylene modified bituminous binders: advantages and challenges. *Road Materials and Pavement Design*, *24*(2), 311-339. https://doi.org/10.1080/14680629.2021.2017330.

Wang, D., Baliello, A., Poulikakos, L., Vasconcelos, K., Kakar, M. R., Giancontieri, G., ... & Falchetto, A. C. (2022). Rheological properties of asphalt binder modified with waste polyethylene: An interlaboratory research from the RILEM TC WMR. *Resources, Conservation and Recycling*, *186*, 106564. https://doi.org/10.1016/j.resconrec.2022.106564.

Zhu, X., Miljković, M., Li, R., Hao, G., Chen, X., & Wang, Y. (2025). Non-linear viscoelastic and micromorphological properties of SBS-modified asphalt binder. *Materials & Design*, *253*, 113958. https://doi.org/10.1016/j.matdes.2025.113958.

**Disclaimer/Publisher's Note:** The statements, opinions and data contained in all publications are solely those of the individual author(s) and contributor(s) and not of MDPI and/or the editor(s). MDPI and/or the editor(s) disclaim responsibility for any injury to people or property resulting from any ideas, methods, instructions or products referred to in the content.
